# Geographical and Climatic Variation Is Associated with Essential Oil Chemotypes While Preserving Biological Activity in *Athamanta sicula* L.

**DOI:** 10.3390/plants15192999

**Published:** 2026-09-30

**Authors:** Giusy Castagliuolo, Alessia Sordillo, Mario Varcamonti, Antonella Porrello, Natale Badalamenti, Giuseppe Bazan, Maurizio Bruno, Anna Zanfardino

**Affiliations:** 1 Department of Biology, University of Naples Federico II, 80126 Naples, Italyalessiasordillo2015@gmail.com (A.S.); varcamon@unina.it (M.V.);; 2 Department of Biological, Chemical and Pharmaceutical Sciences and Technologies (STEBICEF), Università degli Studi di Palermo, Viale delle Scienze, Ed. 17, 90128 Palermo, Italymaurizio.bruno@unipa.it (M.B.)

**Keywords:** *Athamanta sicula* L., chemotype, phytochemical differentiation, apiol, myristicin, antimicrobial activity

## Abstract

Several species of the genus *Athamanta* L. (Apiaceae) have been used since ancient times for their remarkable therapeutic properties and biological potential. Among them, *Athamanta sicula* L. is the only species of this genus found in Sicily, Italy. This study highlights the chemotaxonomic variability of the essential oils of this species by correlating chemical results with environmental and geographical data. Chemical analysis by GC-MS confirmed the predominance of apiol (47.5%), while spectroscopic analysis confirmed both the spectrometric characterization of the entire essential oil and the analytical purity of the isolated apiol. Biological assays showed that the apiol-rich essential oil retained antimicrobial and antibiofilm activities comparable to those of the previously characterized myristicin-rich chemotype, while displaying enhanced activity against *Pseudomonas aeruginosa*. Membrane-permeabilization assays supported membrane damage as a major antibacterial mechanism, whereas antioxidant tests revealed a more efficient scavenging response of the apiol-rich chemotype at lower concentrations. Overall, these findings suggest that climate-associated chemotypic variation modifies specific functional traits without substantially compromising the biological activity of the essential oil.

## 1. Introduction

The genus *Athamanta* L. (Apiaceae) has undergone substantial taxonomic revision over time, resulting in a complicated nomenclatural history. Numerous taxa that were once classified within *Athamanta* have subsequently been reassigned to other genera, including *Seseli* L., *Peucedanum* L., *Libanotis* Haller ex Zinn, *Daucus* L., and *Cenolophium* W.D.J. Koch, among others. According to Plants of the World Online [1], the genus currently comprises only sixteen accepted species distributed from the Canary Islands across central Europe to the Mediterranean region [1].

The medicinal value of *Athamanta* species has been recognized since antiquity. In *De Materia Medica* [2] seeds and roots of *Athamanta cretensis* L. are used as therapeutic agents employed for their antidote properties and for the treatment of a wide range of conditions [2]. Today, *A. cretensis* continues to play a role in traditional Persian medicine, where it is included in herbal preparations intended to promote maternal health, support uterine and reproductive functions, and reduce inflammation [3]. In addition, both *A. cretensis* and *Athamanta macedonica* (L.) Spreng. have been traditionally used in several European regions as folk remedies for sclerosis and cancer [4].

Although historical botanical literature does not attribute medicinal properties to this species, therapeutic applications have been documented for other representatives of the *Athamanta* genus, particularly *Athamanta meum* L. (currently accepted as *Meum athamanticum* Jacq.). Mattioli [5] reported that the roots of this plant, administered either after boiling or in their fresh crushed form, were traditionally employed as a diuretic to relieve kidney and bladder obstructions, as a carminative to improve digestive disorders, as an analgesic for general body pain, and as an emmenagogue to treat gynecological conditions.

To date, investigations on the non-volatile phytochemical constituents of *Athamanta* species remain scarce. Available studies include analyses of the fatty acid composition of fixed oils extracted from the fruits of *Athamanta turbith* subsp. *hungarica* (Borbás) Tutin, and *A. turbith* subsp. *haynaldii* (Borbás & Uechtr.) Tutin [6], the isolation of sesquiterpenes, phenylpropanoids, sterols, triterpenes, diterpene derivatives, and polyacetylenic compounds from *Athamanta vayredana* (Font Quer) C. Pardo aerial parts [7,8], and the identification of phthalides in the roots of *Athamanta montana* (Webb ex Christ) Spalik, Wojew., & S.R. Downie [9]. A study by Aggoun et al. [10] further demonstrated that polar extracts obtained from *A. sicula* aerial parts, characterized primarily by phenolic acids, exhibited notable antioxidant capacity, significant anti-diabetic potential, pancreatic lipase inhibition, strong protection against protein denaturation, and moderate cytotoxic effects on breast cancer cell lines.

Although *Athamanta* species have long been used in traditional medicine to treat renal, dermatological, and gastrointestinal disorders, current research is increasingly focused on their antimicrobial potential in response to the global rise of antimicrobial resistance. The growing prevalence of multidrug-resistant pathogens has intensified the search for new antimicrobial agents with novel mechanisms of action [10]. Natural products, particularly essential oils (EOs), have attracted considerable attention because they target multiple microbial processes, including membrane integrity, biofilm formation, quorum sensing, etc. [11,12,13]. Numerous studies have reported significant antimicrobial activity of EOs against several pathogens, and Apiaceae Eos, rich in monoterpenes and phenylpropanoids, have shown promising activity against uropathogens such as *Escherichia coli* and *Enterococcus* spp., supporting their potential relevance in the treatment of renal-associated infections [12,14,15,16].

The work here proposed concerns the study of *Athamanta sicula* L., the only representative of the genus in Sicily. Based on POWO [1], its native range includes Sicily, the Italian Peninsula, and Nord-West Africa. The species typically inhabits calcareous rocky habitats, including limestone cliffs and ancient masonry walls, occurring from coastal areas up to approximately 1500 m above sea level [17,18]. *A. sicula* occupies a recognized place in Sicilian traditional medicine, where it is primarily valued for its diuretic activity and its reputed ability to promote the expulsion of renal calculi [19,20].

Although secondary metabolite production is largely determined by the plant genotype, environmental conditions are also recognized as key drivers of their biosynthesis and chemical composition, particularly in the case of EOs [21,22,23,24,25]. A wide range of environmental stressors, including temperature extremes, water deficit, salinity, heavy metals and metalloids, UV-B radiation, and ozone exposure, have been shown to affect both the yield and chemical profile of EOs by altering the relative abundance of their major constituents [26,27,28,29,30,31,32,33].

Water availability likewise plays a pivotal role in determining EO production. Drought stress has been reported to influence not only EO yield but also its chemical composition and the regulation of the underlying biosynthetic pathways. Current evidence indicates that water deficit can induce quantitative changes in individual terpenes and phenylpropanoids while also modulating the expression of genes involved in their biosynthesis [34,35,36,37,38]. These changes are likely to contribute to plant acclimation to water-limited environments by reshaping the chemical profile of EOs according to the intensity and duration of drought stress.

Quantitative and qualitative variation in EOs among populations of the same species is closely associated with their geographic distribution. In particular, latitudinal and altitudinal gradients generate contrasting climatic regimes and edaphic conditions that act as selective environmental drivers, modulating the biosynthesis of volatile metabolites and consequently influencing both the yield and chemical profile of EOs [39,40,41,42].

Rupicolous species inhabit ecologically extreme and highly heterogeneous environments, where they are exposed to multiple and often interacting environmental stresses. In these habitats, lithology plays a major role in shaping local ecological conditions by influencing water availability, nutrient supply, metal content, substrate depth and temperature, thereby exerting strong selective pressures on plant physiology and secondary metabolism [43,44,45,46,47].

**Figure 1:**
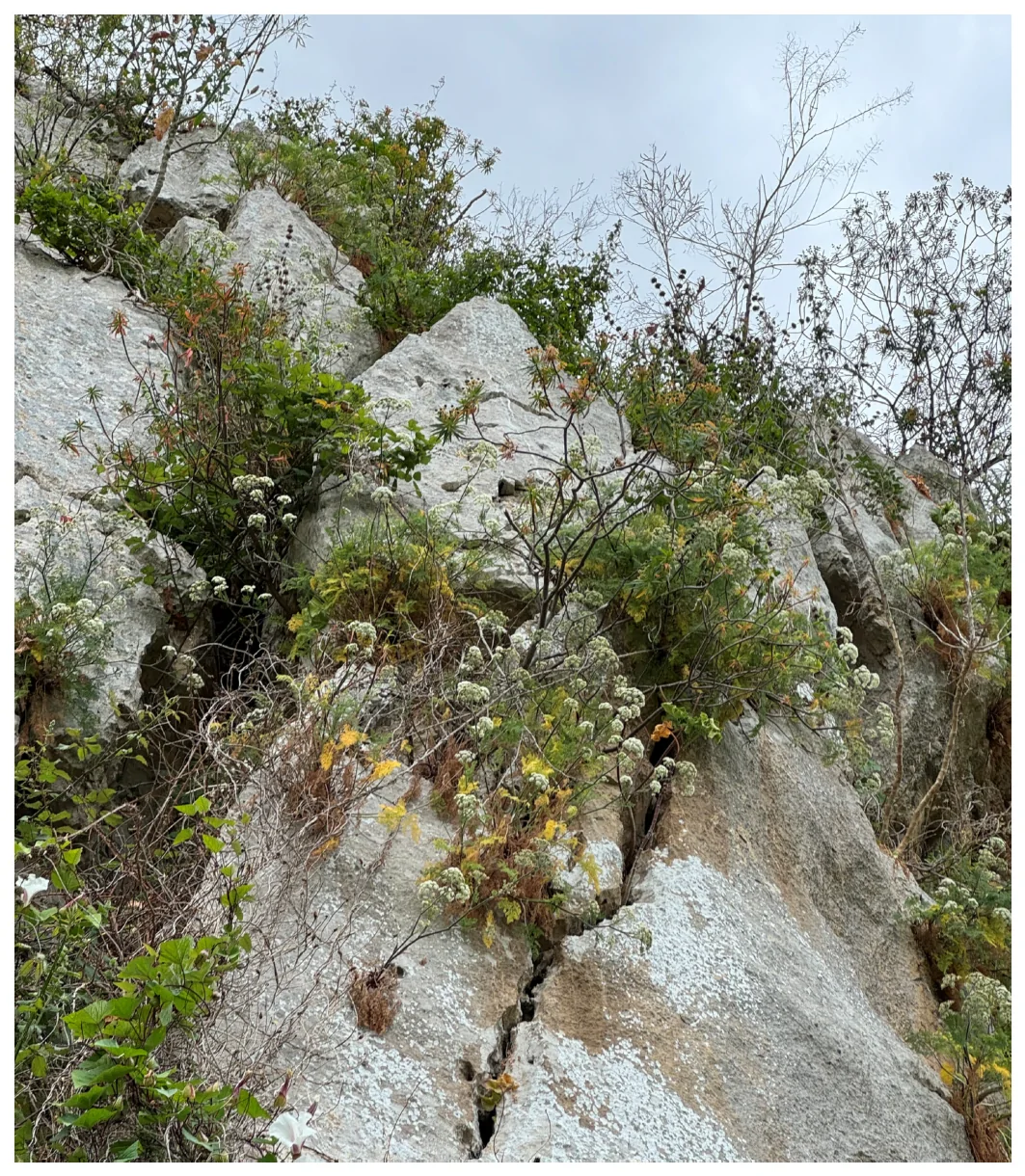
*Athamanta sicula* L. collected at Roccamena, Sicily, Italy. Photo by Giuseppe Bazan.

Previously investigation on the EOs from the aerial parts of *A. sicula* collected in Algeria [48], and Sicily [49,50,51] showed the presence of three different chemotypes: germacrene B [48], myristicin [49,50], and apiol [51] chemotypes. In the present communication the chemical composition of the EO of another accession of *A. sicula,* collected at Roccamena (Sicily) (Figure 1), is reported. Furthermore, antimicrobial, antibiofilm, and antioxidant effects of this EO have been determined and the relationship between climatic conditions and the occurrence of different chemotypes has been explored.

## 2. Results and Discussion

### 2.1. Chemical Composition of A. sicula Essential Oil by GC-MS and 1D-NMR

Hydrodistillation of *A. sicula* aerial parts, collected at full flowering stage, gave a yellow EO (**As**). Overall, twenty-three compounds were identified, representing 97.3% of total composition, listed in Table 1 according to their retention indices on a DB-5 MS column and classified into four chemical classes. Phenyl propanoids represented the most abundant class (48.3%) with apiol (47.5%), by far, as the principal metabolites of the class and of the entire sample. Monoterpene hydrocarbons formed the second main class, representing 45.3% of the total, with *β*-phellandrene (32.7%) and *γ*-terpinene (6.3%) as the most abundant components. Sesquiterpene hydrocarbons occurred in quite poor quantity (3.3%) and oxygenated terpenoids, both monoterpenic and sesquiterpenic derivatives, were practically absent.

**Figure 2:**
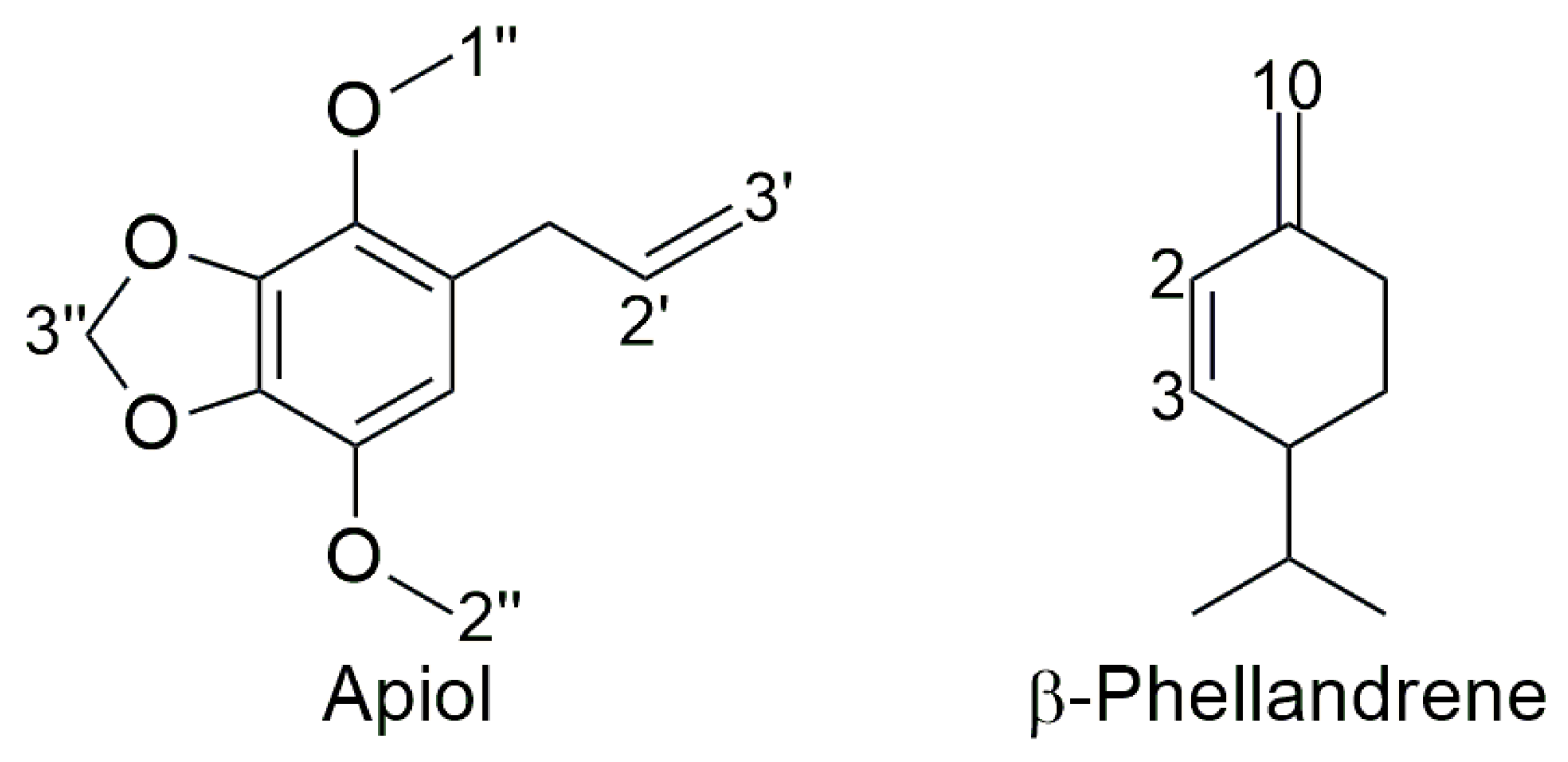
Chemical structure of *β*-phellandrene and apiol.

The phytochemical identification of the **As** major compounds (*β*-phellandrene and apiol), carried out by chromatographic analysis, was also confirmed by means of the NMR spectroscopic technique, in particular by exploiting ^1^H-NMR spectrum. *β*-Phellandrene and apiol, the first one is hydrocarbon monoterpene and the other a phenylpropanoid, they possess different chemical structures (Figure 2), but both are characterized by distinctive exocyclic protons.

**Figure 3:**
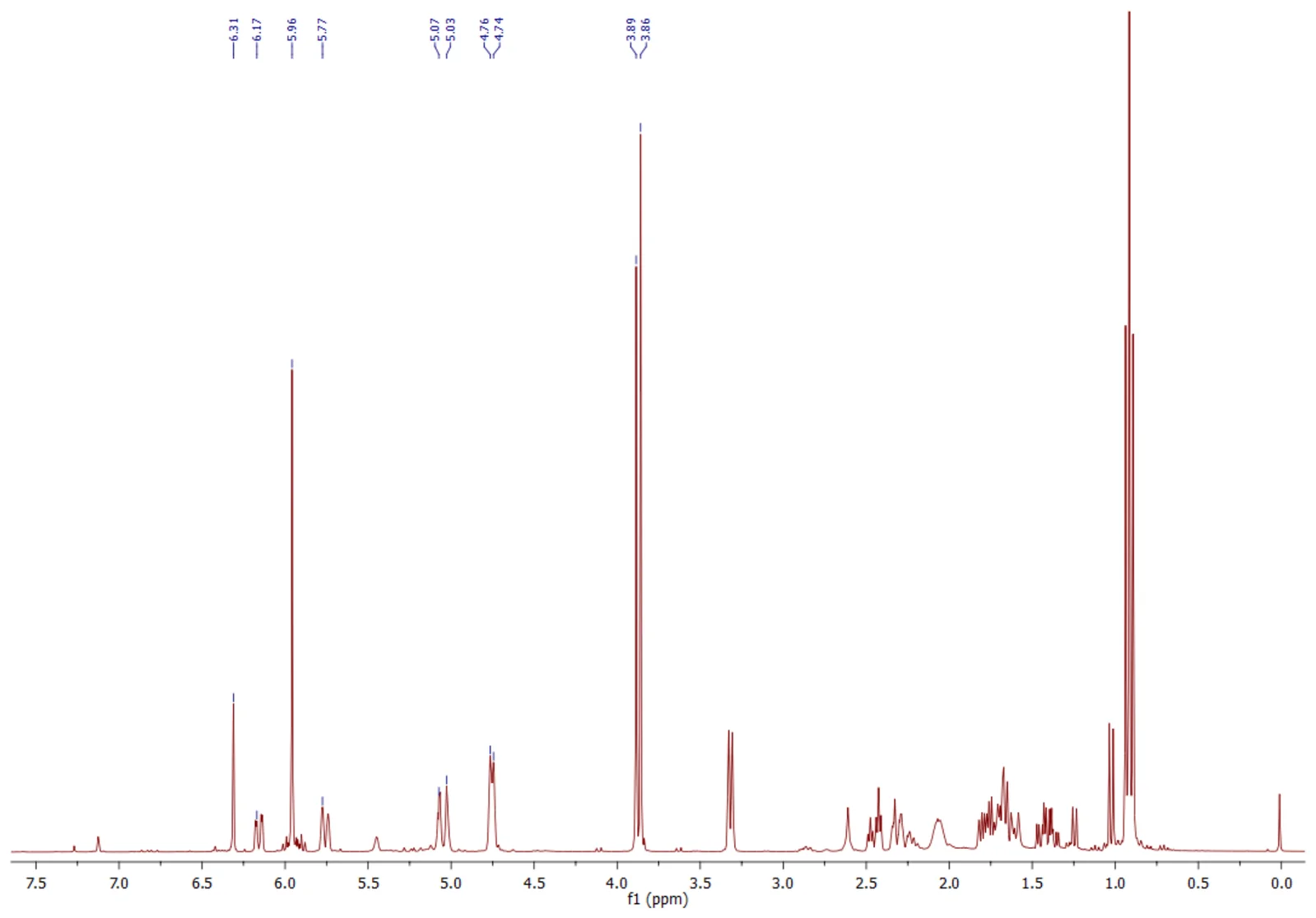
^1^H-NMR spectrum of *Athamantha sicula* EO (**As**).

The signals for these groups—2H-10 for phellandrene and 2H-3′ for apiol—are easily identified in the ^1^H-NMR spectrum (Figure 3): the former appear as two distinct signals at 5.03 and 5.07 ppm, while the latter overlap in the 4.74–4.76 ppm range. Apiol also exhibits three other characteristic signals: an aromatic proton on the pentasubstituted benzene ring (6.31 ppm), a methylenedioxy group at 5.96 ppm, and finally two singlet signals for -OCH_3_ groups at 3.86 and 3.89 ppm. In contrast, the endocyclic protons H-2 and H-3 of *β*-phellandrene exhibit characteristic peaks at 5.77 and 6.17: the first signal—a doublet with a coupling constant (*J*) of 10.3 Hz—is attributable to H-2, while the doublet of doublets at 6.17 ppm corresponds to H-3 proton. The spectroscopic analysis thus confirmed the data obtained via GC-MS and, above all, validated the structural identification of the major compounds in this EO.

### 2.2. Relationships Between Environmental Variables and Volatile Compound Abundance

The five populations of *A. sicula* differed markedly in both their bioclimatic setting and their volatile chemical profiles. To explore whether these two sources of variation were related, climatic variables, MaxEnt [52] suitability and the relative abundance of the main volatile compounds were examined together across sites. The sampled sites showed clear climatic differentiation (Table 2).

**Figure 4:**
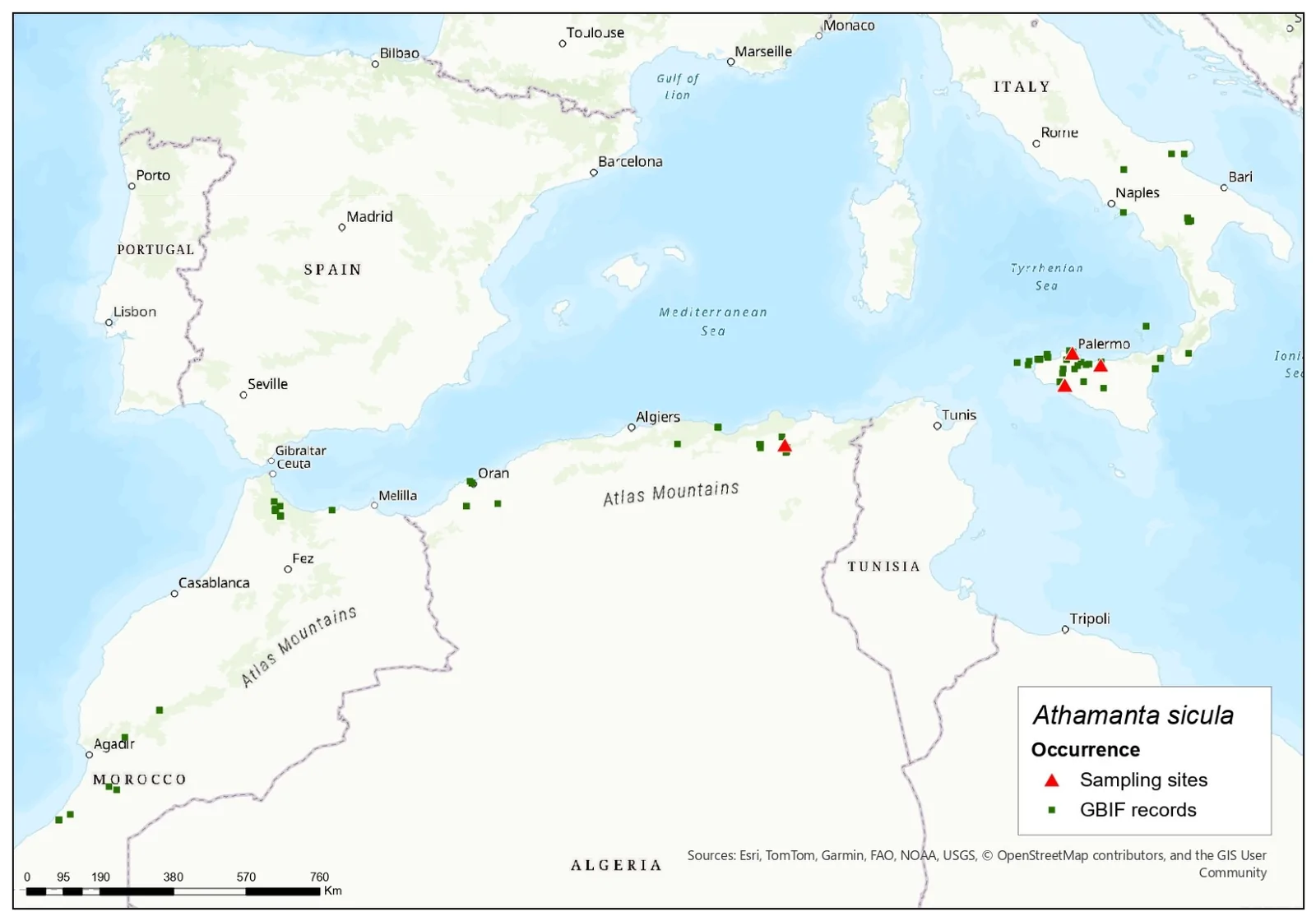
Climatic suitability map for *A. sicula* generated using MaxEnt v.3.4.1. The model was calibrated using occurrence records of the species and eight continuous bioclimatic variables derived from CHELSA. Suitability values represent the relative climatic suitability predicted by the model, with higher values indicating areas with climatic conditions more suitable for the potential occurrence of the species. Bioclimatic variables correspond to CHELSA V2.1 climatological means for the period 1981–2010 at 30 arc-second (~1 km) resolution.

Roccamena showed the highest annual mean temperature (BIO1 = 16.95 °C), while Monte Pellegrino was characterized by the highest minimum temperature of the coldest month (BIO6 = 7.75 °C), indicating the mildest winter conditions among the sampled sites; in contrast, the Madonie site showed the lowest annual mean temperature (BIO1 = 11.15 °C) and the highest annual precipitation (BIO12 = 928 mm). Didouche Mourad was distinguished by the highest maximum temperature of the warmest month (BIO5 = 31.65 °C), the highest temperature seasonality (BIO4 = 6.67), and one of the lowest suitability values (0.35), shared with Roccamena. In contrast, Caltabellotta, Monte Pellegrino, and Madonie showed very high suitability values, ranging from 0.99 to 1.00, indicating that these sites fall within the core climatic suitability space predicted for the species (Figure 4).

The chemical profiles also showed strong differentiation among populations (Table 3). Apiol was clearly dominant in the Monte Pellegrino population, where it reached 84.47% of the selected volatile fraction, and it was also abundant in Roccamena, where it reached 47.5%. It occurred at much lower values in Caltabellotta and Didouche Mourad and was absent in Madonie. Myristicin showed an opposite pattern, with very high values in Madonie (97.6%) and Caltabellotta (62.2%), low abundance in Monte Pellegrino (4.5%), and absence in Roccamena and only trace amounts in Didouche Mourad (0.4%). Germacrene B was almost exclusively associated with the Didouche Mourad population, where it reached 88.5%. *β*-Phellandrene was detected in both Roccamena (32.7%) and Caltabellotta (12.2%), whereas *γ*-terpinene occurred at low values in Caltabellotta (6.9%) and Roccamena (6.3%).

The exploratory correlation analysis highlighted marked site-level differences in the association between climatic conditions, MaxEnt suitability and the relative abundance of the main volatile compounds detected in the investigated populations of *A. sicula* (Table 4).

**Figure 5:**
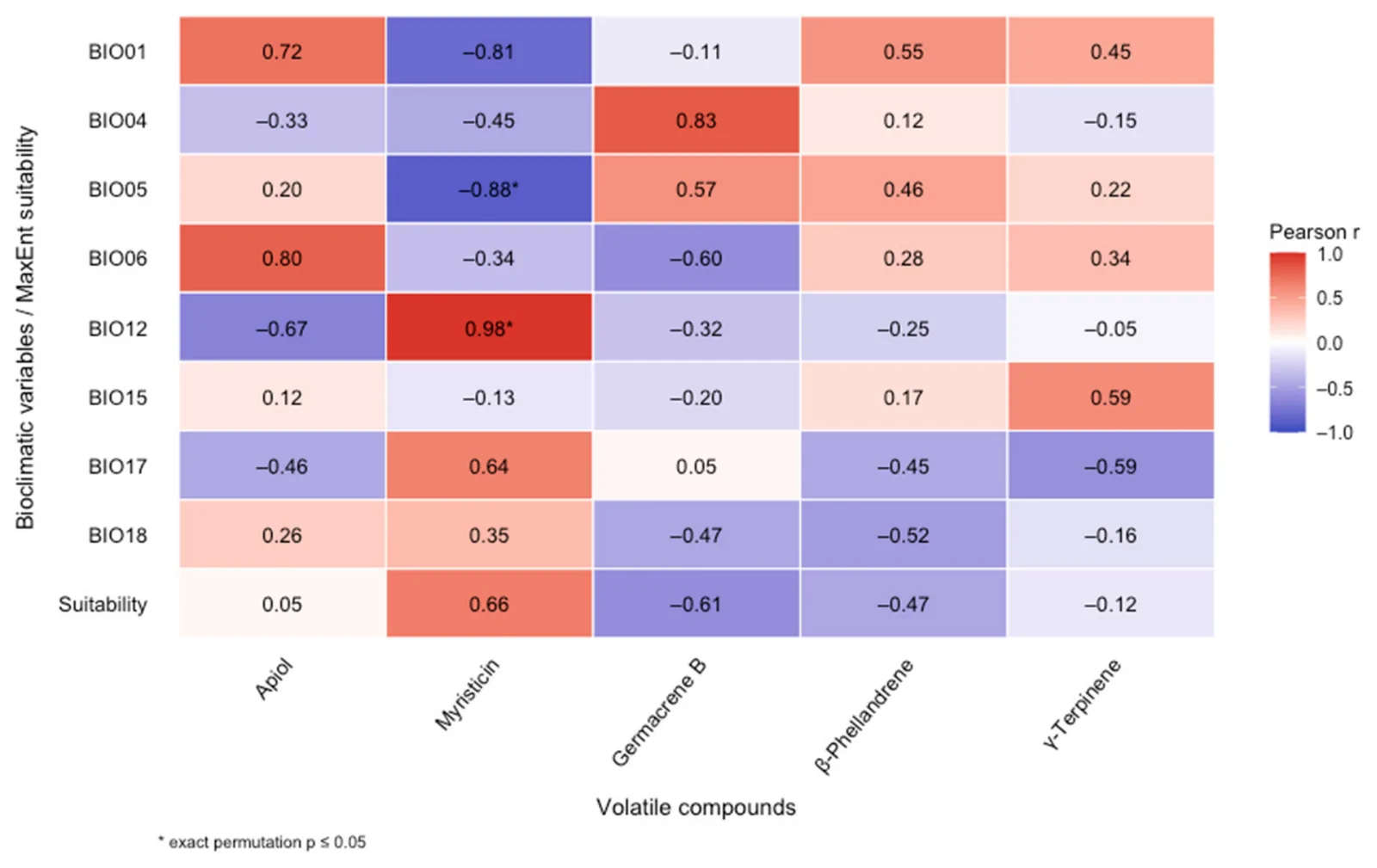
Heatmap of Pearson correlation coefficients between bioclimatic variables, MaxEnt suitability and the relative abundance of the main volatile compounds detected in the sampled populations of *Athamanta sicula*. BIO01 = annual mean temperature (°C); BIO04 = temperature seasonality; BIO05 = maximum temperature of the warmest month (°C); BIO06 = minimum temperature of the coldest month (°C); BIO12 = annual precipitation (mm); BIO15 = precipitation seasonality; BIO17 = precipitation of the driest quarter (mm month^−1^); BIO18 = precipitation of the warmest quarter (mm month^−1^); Suitability = climatic suitability value estimated by the MaxEnt model. Bioclimatic variables are CHELSA V2.1 climatological means for the period 1981–2010 [53,55].

Given the limited number of sampled sites, the correlation coefficients were interpreted primarily in terms of direction and magnitude of association, rather than as formal evidence of statistical significance. Accordingly, Pearson’s correlation coefficient (Figure 5), Spearman’s rank correlation coefficient and Kendall’s tau were used as complementary descriptive metrics to explore potential ecological trends.

Apiol showed a moderate to strong positive association with thermal variables, particularly with the minimum temperature of the coldest month (BIO6; Pearson’s r = 0.802) and annual mean temperature (BIO1; Pearson’s r = 0.718). The rank-based correlation with BIO1 was also very high (Spearman’s ρ = 0.800; Kendall’s τ = 0.600), indicating that the ordering of the sites by annual mean temperature corresponded closely to the ordering of sites by apiol abundance. Conversely, apiol was negatively associated with annual precipitation (BIO12; Pearson’s r = −0.670; Spearman’s ρ = −1.000; Kendall’s τ = −1.000). This pattern suggests that, among the Sicilian populations, apiol tends to be more abundant at sites with relatively warm conditions, and with comparatively lower annual pre-capitation. This is reflected in its association with annual mean temperature (BIO1) and with the minimum temperature of the coldest month (BIO6), rather than with the maxi-mum temperature of the warmest month (BIO5). Monte Pellegrino and Roccamena are the two main apiol-rich populations, although their individual thermal profiles differ, whereas wetter sites such as Madonie are characterized by low or absent Apiol. This association does not extend to the North African population of Didouche Mourad, which, despite its dry and thermally seasonal climate, shows very low apiol abundance and a distinct, germacrene B–dominated chemical profile. Overall, these observations support the hypothesis of an apiol-rich chemical profile associated with the milder, relatively warmer and drier Sicilian populations.

Myristicin displayed an opposite relationship with the main climatic gradients. The compound was negatively associated with annual mean temperature (BIO1; Pearson’s r = −0.810) and with the maximum temperature of the warmest month (BIO5; Pearson’s r = −0.880; Spearman’s ρ = −0.975; Kendall’s τ = −0.900). In contrast, myristicin showed a strong positive association with annual precipitation (BIO12; Pearson’s r = 0.982), indicating higher values in cooler and wetter sites. This pattern is consistent with the high abundance of myristicin in Madonie, the coolest and wettest site among those analyzed, and with its relatively high abundance in Caltabellotta. These results suggest a possible climatic differentiation between apiol-rich and myristicin-rich chemotypes, with apiol associated relatively warmer and drier conditions and myristicin associated with cooler and more mesic or humid conditions.

Germacrene B showed a different pattern because its occurrence was almost entirely restricted to the Didouche Mourad population. Consequently, the correlations involving this compound largely reflect the climatic distinctiveness of this single site rather than a continuous response across all populations. Germacrene B was positively associated with temperature seasonality (BIO4; Pearson’s r = 0.832) and with the maximum temperature of the warmest month (BIO5; Pearson’s r = 0.572), while it was negatively associated with the precipitation of the warmest quarter (BIO18; Pearson’s r = −0.466). A negative relationship was also observed with MaxEnt suitability (Pearson’s r = −0.612), reflecting the dominance of germacrene B in one of the two least suitable sites, Didouche Mourad. However, this association should be interpreted with particular caution, as it is driven by a single chemically distinct population.

*β*-Phellandrene and *γ*-terpinene showed partly similar but not identical correlation patterns because both compounds were detected only in Roccamena and Caltabellotta and were absent from the other sites. *β*-Phellandrene showed a moderate positive association with annual mean temperature (BIO1; Pearson’s r = 0.554) and a weak negative relationship with annual precipitation (BIO12; Pearson’s r = −0.247). *γ*-Terpinene showed a moderate positive association with precipitation seasonality (BIO15; Pearson’s r = 0.593) and a negative association with precipitation of the driest quarter (BIO17; Pearson’s r = −0.588). Nevertheless, due to their restricted occurrence in only two sites (Roccamena and Caltabellotta), these correlations should be regarded as indicators of local chemical specificity rather than evidence of a general climatic response.

The relationship between MaxEnt suitability and volatile compounds was generally weak or moderate for most metabolites. Apiol showed a very weak positive correlation with suitability (Pearson’s r = 0.045), while myristicin showed a moderate positive correlation (Pearson’s r = 0.663). *β*-Phellandrene showed a moderate negative correlation with suitability (Pearson’s r = −0.469), whereas *γ*-terpinene showed a weak negative relationship (Pearson’s r = −0.124). Germacrene B showed a moderate negative relationship with suitability (Pearson’s r = −0.612), reflecting its dominance in one of the two least suitable sites (Didouche Mourad). This suggests that the main suitability gradient identified by MaxEnt may not directly explain the abundance of all volatile compounds, but it may help distinguish populations occurring within the core climatic niche from those located under more marginal climatic conditions.

Only a few associations approached or reached conventional statistical significance when evaluated using exact *p*-values, and these results should still be interpreted with caution because of the very small sample size. The clearest pattern involved myristicin and BIO5, with a strong negative correlation (Pearson’s r = −0.880; *p* = 0.050; Spearman’s ρ = −0.975; *p* = 0.033), suggesting that myristicin decreases with increasing maximum temperature of the warmest month. Apiol also showed a strong negative monotonic relationship with annual precipitation (BIO12; Spearman’s ρ = −1.000; *p* = 0.017; Kendall’s τ = −1.000), indicating that the apiol-rich populations occur toward the drier end of the climatic gradient. However, because the analysis is based on only five populations, these results should be considered exploratory and hypothesis-generating rather than conclusive. Despite this limitation, the consistency of some patterns across Pearson, Spearman and Kendall coefficients suggests a possible ecological signal that warrants testing on a larger sample, particularly the contrast between apiol-rich populations, which are associated with relatively warmer conditions and comparatively lower precipitation, and myristicin-rich populations, which are associated with cooler and wetter conditions.

Taken together, these preliminary results indicate that the chemical variability observed among populations of *Athamanta sicula* may be partly related to climatic differentiation among sites.

It should be noted that the bioclimatic variables used in this study are long-term climatological means (1981–2010). They therefore characterize the climatic regime to which each population has been exposed over time, i.e., the environmental context in which selective pressures on chemotypic composition may have operated, rather than the weather conditions experienced by the plants during the growing season preceding sampling. Interannual weather variability can nevertheless modulate EO yield and the relative proportions of individual constituents, particularly through water deficit and temperature anomalies [34,35,36,37,38]. This aspect is relevant to the present comparison, since the Roccamena population was sampled on 2 May 2024 during an exceptionally dry period in Sicily: the regional mean precipitation accumulated between June 2023 and May 2024 was about 453 mm, the lowest value recorded since the severe drought of 2002 [56]. This twelve-month regional statistic provides context and extends beyond the sampling date.

To assess the weather conditions of the growing seasons preceding sampling, daily temperature and precipitation were retrieved from the ERA5 reanalysis [57] through Open-Meteo [58] for the nearest ERA5 land grid point to each site and compared with ERA5 means for the corresponding calendar-day interval over 1981–2010, i.e., the same reference period as the CHELSA climatologies (see Section 3.1). At Roccamena, the period from 1 September 2023 to the sampling date was markedly drier (410 vs. 536 mm; −24%, the seventh driest of the 45 seasons between 1981 and 2025) and warmer (+1.8 °C) than the reference period, whereas at Caltabellotta the period from 1 September 2024 to the sampling date (10 May 2025) received near-normal precipitation (445 vs. 441 mm), although it was warmer (+1.2 °C). However, the same analysis applied to the literature populations, although based on approximate sampling dates, indicates that the apiol-rich Monte Pellegrino population was sampled after a wetter-than-average season (+15%), whereas the myristicin-rich Madonie population was sampled after a drier-than-average one (−14%). The occurrence of both chemotypes under contrasting seasonal conditions does not support a simple correspondence between the precipitation anomaly of the sampling season and the dominance of apiol or myristicin. Drought-related changes in EO composition can affect the relative abundance of individual constituents [34,35,36,37,38], whereas the contrast between the profiles examined here involves a change in the dominant phenylpropanoid, the alternative one being absent or only a minor constituent (0–4.5%). This is consistent with the occurrence of distinct chemotypes whose expression may be modulated by seasonal weather but does not by itself establish a genetic basis or exclude environmental effects on constituent dominance. Multi-year sampling of the same populations will be required to assess temporal stability and disentangle the effects of long-term climate from those of interannual weather variability.

A further source of uncertainty is the heterogeneity of the chemical data, which were obtained over a period of 25 years (2000–2025), in different months (May–July) and from different plant organs [48,49,50,51]. Nevertheless, within the literature populations the dominant phenylpropanoid was consistent across the organs analyzed: apiol prevailed in the flower, stem and leaf EOs of the Monte Pellegrino population (80.4–87.8%) [51], whereas myristicin was the main constituent of both the leaf (97.6%) and the fruit (41.3%) EOs of the Madonie population, in which apiol did not exceed 0.5% [49]. These observations support a distinction between population-associated chemotypic profiles across the organs examined, but do not establish independence from organ or sampling period. The relative abundance of the main constituents clearly varies among organs, as shown by the markedly different leaf and fruit EOs of the Madonie population [49]. A similar interpretation was already proposed by Camarda et al. [51], who suggested that the differences between the Monte Pellegrino and Madonie samples could be explained either by different habitat conditions or by the occurrence of distinct chemotypes.

Beyond environmental and ontogenetic factors, the genetic component of chemical variation must also be considered. Genetically based differences in EO composition can coexist with effects of environmental conditions, developmental stage and plant organ on constituent proportions [26]. In *Thymus vulgaris* L., for instance, the chemical polymorphism is genetically determined, yet the spatial distribution of the chemotypes tracks climatic gradients and has shifted in association with changes in extreme winter freezing events [59]. In such cases, climate can act as a selective factor on genetically differentiated chemotypes, as well as potentially influencing their chemical expression. This provides a hypothesis for *A. sicula*, for which the available data do not yet allow these components to be disentangled.

Karyological studies have consistently reported 2*n* = 22 for Sicilian populations of *A. sicula* from Chiusa Sclafani, Erice, Forza d’Agrò and Palermo, as well as for material from the Italian Peninsula [60,61,62,63], indicating no chromosome-number differentiation among the populations examined so far. These counts do not exclude genetic differentiation at the sequence or regulatory level. Notably, Colombo and Marcenò [62] described the population from contrada Muscola, in the Chiusa Sclafani area, about 15 km from the Caltabellotta site, as morphologically distinct, with slenderer individuals and broader fourth-order leaf segments. Such observations motivate further investigation of intraspecific variation, but do not establish a link to the chemotypes analyzed here.

The molecular studies considered here included single accessions of Moroccan origin: nuclear ribosomal ITS sequences analyzed as *Tinguarra sicula* in a tribal phylogeny [64], and an accession of *A. sicula* used as an outgroup in a study of *Daucus* based on ten nuclear orthologues [65]. Combined analyses of morphology, fruit anatomy and ITS sequences [54,66] supported the inclusion of *Tinguarra* Parl. in *Athamanta*. These studies provide no information on intraspecific genetic structure in *A. sicula*. To our knowledge, no published population genetic or phylogeographic study, nor a common-garden experiment comparing different populations, has yet tested the basis of chemotypic differentiation in this species. Consequently, the chemotypic differentiation documented here may reflect genetic differentiation (possibly driven by local adaptation), phenotypic plasticity, or their interaction, and should be interpreted as a pattern of association rather than as evidence of a direct climatic effect. Integrating population genomic data with repeated chemical sampling and common-garden cultivation of plants from chemically characterized populations would allow these hypotheses to be tested.

### 2.3. Antimicrobial Activity and Comparison Between Athamanta sicula Chemotypes

In light of the marked chemical differentiation observed among the *Athamanta sicula* populations, it was considered of particular interest to compare the apiol-rich EO (**As**) from Roccamena investigated in the present study with the myristicin-rich (**Ca**) previously described for the Caltabellotta population [50]. The choice of this comparison was primarily motivated by the pronounced differences in their volatile profiles despite the relatively similar broad bioclimatic setting of the two populations. Indeed, both Roccamena and Caltabellotta fall within the Lower Mesomediterranean thermotype and the Lower Subhumid ombrotype, although they differ in their predicted climatic suitability according to the MaxEnt model (0.35 and 1.00, respectively). Therefore, the population of Caltabellotta represents the most appropriate reference for a direct comparison of biological activity because the antimicrobial assays were performed by our group using experimental procedures comparable to those applied in the present study. This methodological consistency allows differences in MIC values to be interpreted more reliably than comparisons with studies employing different antimicrobial susceptibility testing methods. The comparison is also of phytochemical interest, since the two EOs display markedly different predominant phenylpropanoids despite the relatively similar broad bioclimatic setting of the two populations.

As shown in Table 5, the results indicate that both samples possess interesting antimicrobial activity against the selected bacterial strains, with MIC values generally ranging between 17 and 30 mg/mL.

Overall, the differences observed between the two oils were relatively limited. **As** showed greater efficacy against *P. aeruginosa* and *B. subtilis*, with MIC values of 17 and 23 mg/mL, respectively, compared to the values of 25 and 26 mg/mL observed for **Ca**. Conversely, the myristicin-rich sample was slightly more active against *S. Typhimurium* and *L. monocytogenes*. For *E. coli*, *S. aureus*, and *M. smegmatis*, the MIC values were substantially overlapping. These results suggest that the substitution of myristicin with apiol does not lead to substantial changes in the overall antibacterial potential of the phytocomplex, except against *P. aeruginosa*, where the highest potential of **As** was observed. It is therefore plausible that the observed activity derives from the combined action of the major phenylpropanoids and other metabolites present in **As**, rather than from the concentration of the main compound alone.

Previous investigations on *A. sicula* also support the occurrence of antibacterial activity across chemically distinct EOs. In particular, Labed et al. [48] investigated an EO obtained from an Algerian population of *A. sicula*, characterized by a markedly different phytochemical profile, with germacrene B (88.5%) as the predominant constituent and apiol accounting for only 4.9% of the oil. Despite this substantial compositional difference, the Algerian EO exhibited antibacterial activity against several Gram-positive and Gram-negative bacteria, including *E. coli*, *S. aureus*, and *P. aeruginosa* (10–18 μg/mL) [48]. Interestingly, these microorganisms were also susceptible to the Roccamena and Caltabellotta EOs investigated in the present and previous studies [50].

A direct quantitative comparison of the antimicrobial potency should, however, be avoided because Labed et al. [48] evaluated antibacterial susceptibility using disk diffusion and agar dilution methods, whereas the present study and our previous investigation on the Caltabellotta chemotype [50] employed broth microdilution under comparable experimental conditions. For this reason, the **Ca** represents the most appropriate reference for a direct comparison of MIC values. Nevertheless, the activity reported for the germacrene B-rich Algerian population provides an interesting broader context, suggesting that antibacterial activity may be retained across markedly different *A. sicula* chemical profiles.

Taken together, the available evidence indicates that the antibacterial properties of *A. sicula* are not restricted to a single chemotype or predominant constituent. Rather, different phytochemical profiles, dominated by germacrene B, myristicin, or apiol, may retain antibacterial activity, although the magnitude and spectrum of this activity may vary according to EO composition. This observation further supports the interpretation of *A. sicula* EO as a complex phytochemical system in which both major constituents and interactions with minor components may contribute to the overall antimicrobial effect.

To further investigate the possible contribution of the predominant metabolites, the antimicrobial activity of pure apiol was subsequently evaluated and compared with that of myristicin, the results of which are shown in Table 6.

The analysis of the isolated substances revealed more marked differences than those observed for the whole EOs. In particular, apiol showed lower MIC values than myristicin against most of the tested microorganisms. The most evident differences were observed against *E. coli* (10 vs. 15 mg/mL) and *P. aeruginosa* (9 vs. 14 mg/mL), while substantially comparable activities were detected against *S. aureus*, *B. subtilis* and *S. typhimurium*. Considering that apiol represents over 40% of **As**, these results suggest that this compound plays a decisive role in the antimicrobial activity observed for the new chemotype. Similarly, in **Ca**, myristicin had been previously identified as the main constituent responsible for the biological activity of the EO. In conclusion, the comparison between the two chemotypes highlights that, while maintaining a substantially overlapping spectrum of antibacterial action, the different phytochemical composition reflects variations in efficacy against specific microorganisms. In particular, the apiol-rich chemotype (**As**) shows higher activity against *P. aeruginosa*, both as EOs (MIC = 17 mg/mL vs. 25 mg/mL) and as a pure compounds (9 mg/mL vs. 14 mg/mL), as well as a higher efficacy of apiol compared to myristicin against *E. coli*. These results suggest that the replacement of myristicin with apiol does not substantially alter the overall antibacterial potential of **As**, but it may confer a selective advantage against specific bacterial targets, likely related to the different physicochemical properties of the two phenylpropanoids and their interactions with the minor constituents of the EO.

### 2.4. Effects on the Bacterial Cell Membrane

**Figure 6:**
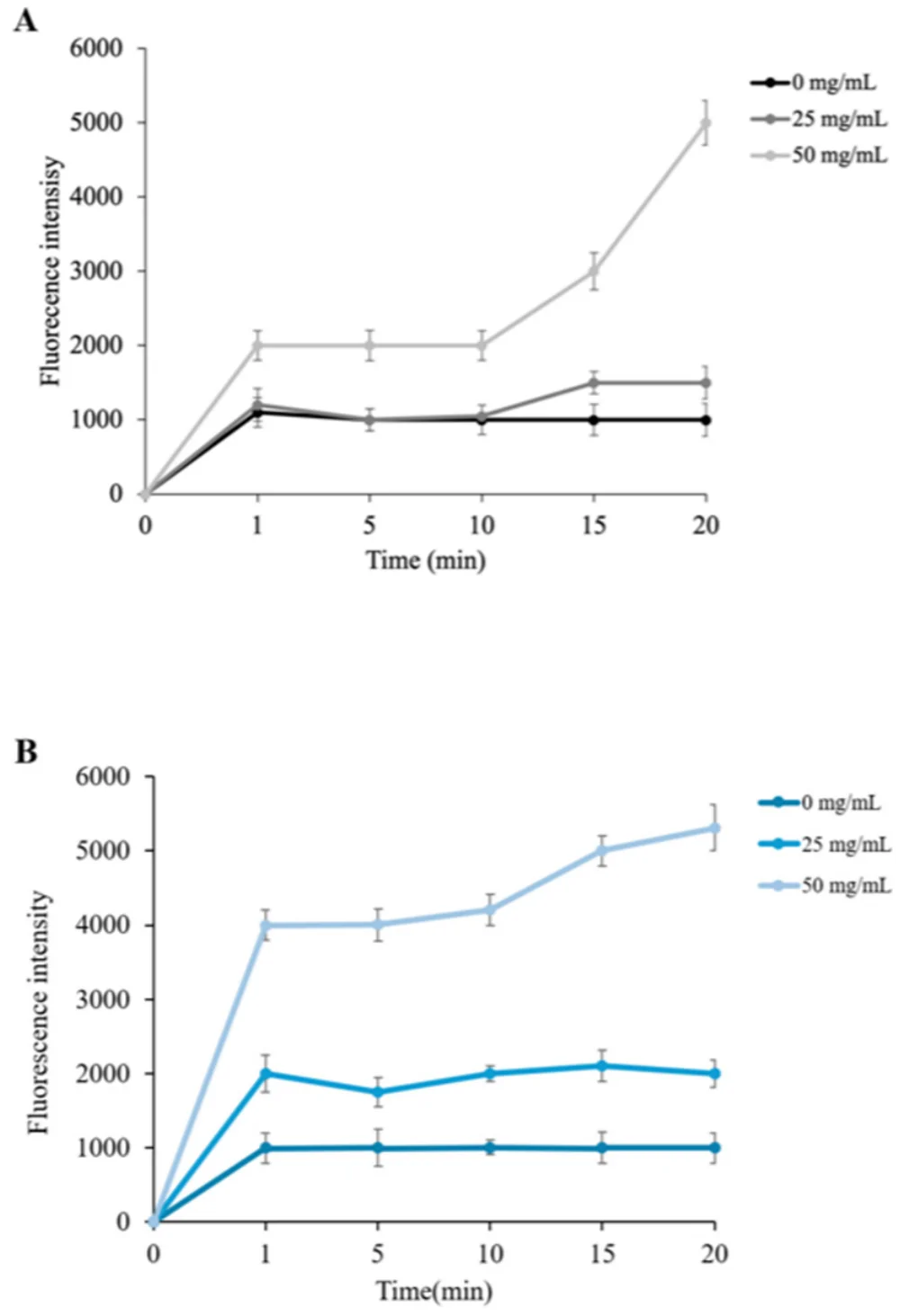
Outer membrane damage assessed by the NPN uptake assay. NPN fluorescence was used to evaluate the outer membrane permeability of *E. coli* after treatment with MIC (25 mg/mL) and 2 × MIC (50 mg/mL) concentrations of **Ca** ((**A**), previously published data), and **As** ((**B**), present study). Data represent the mean ± SD of three independent experiments. **As**: *Athamanta sicula* apiol-rich EO from Roccamena; **Ca**: *A. sicula* myristicin-rich from Caltabellotta.

To obtain information on the potential mechanism of action of the two chemotypes, the ability of both EOs (**As**, and **Ca**) to alter bacterial cell membrane integrity was evaluated using the NPN uptake assay and fluorescence microscopy with DAPI/PI double staining. The NPN assay allows for the monitoring of outer membrane permeabilization in Gram-negative bacteria. Indeed, the fluorochrome N-phenyl-1-naphthylamine exhibits weak fluorescence in an aqueous environment, whereas its emission increases significantly when it penetrates the hydrophobic regions of a damaged membrane. As shown in Figure 6, both chemotypes induced an increase in fluorescence compared to untreated controls, indicating a compromise in bacterial membrane integrity. However, a comparison between the two profiles revealed some differences.

**Figure 7:**
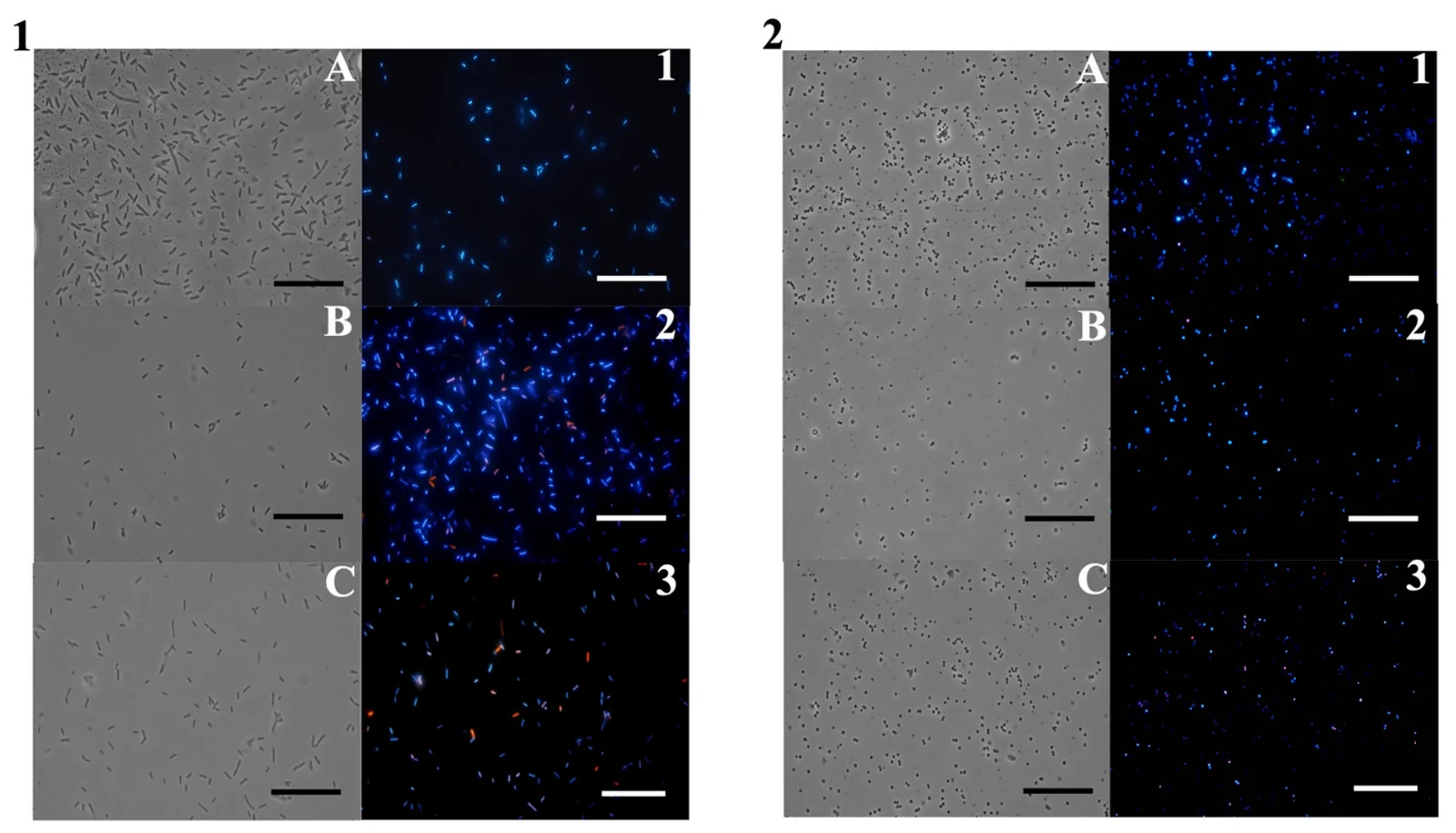
Fluorescence microscopy of *E. coli* (Panel (**1**)) and *S. aureus* (Panel (**2**)) cells after 30 min of treatment with **As** (**B**,**2**) and apiol (**C**,**3**), stained with DAPI (blue) and propidium iodide (red). Untreated control cells are shown in (**A**,**1**). Scale bars: 5 µm.

In **Ca** (Panel A), previously described, treatment at a concentration of 50 mg/mL resulted in a progressive increase in fluorescence, reaching values close to 5000 units after 20 min of incubation, indicating a marked permeabilization of the *E. coli* outer membrane. A similar trend was observed for **As** (Panel B). However, the increase in fluorescence was faster and slightly more pronounced within the first few minutes of treatment, reaching values above 4000 units after one minute of incubation and subsequently exceeding 5000 units. This behavior suggests that the apiol-rich chemotype (**As**) may interact with the bacterial membrane with slightly faster kinetics compared to the myristicin-rich chemotype (**Ca**). To confirm these observations, fluorescence microscopy analyses were performed using DAPI/PI double staining. In this system, DAPI crosses intact membranes and binds to DNA, emitting blue fluorescence, whereas propidium iodide (PI) can exclusively penetrate cells with compromised membranes, resulting in red fluorescence. In the previous study, **Ca** and myristicin had shown a marked presence of red-fluorescent cells in *E. coli*, indicative of a significant loss of membrane integrity [50]. Similar results were also observed for **As** sample (Figure 7).

Specifically, treatment with **As** (B-2) and apiol (C-3) led to the appearance of numerous PI-positive cells in *E. coli* (Panel 1), indicating a marked loss of membrane integrity. Membrane-damaged cells were also observed in *S. aureus* (Panel 2), although to a lesser extent than in the Gram-negative bacterium. The consistency between the NPN uptake assay, which provides evidence of outer membrane permeabilization in *E. coli*, and DAPI/PI staining supports membrane perturbation as an important component of the antibacterial action of **As**.

This finding is particularly relevant when considered in relation to the marked phytochemical divergence between the Roccamena and Caltabellotta chemotypes. In the previously investigated myristicin-rich EO (**Ca**), membrane permeabilization was associated with the high abundance of myristicin, and the isolated compound itself induced a pronounced loss of membrane integrity [50]. In the present study, a comparable membrane-targeting phenotype was observed despite the replacement of myristicin by apiol as the predominant phenylpropanoid. Moreover, isolated apiol also produced PI-positive cells, supporting its contribution to the membrane-damaging effect observed for **As**. Thus, the present results suggest that membrane perturbation may represent a conserved antibacterial mechanism across chemically distinct *A. sicula* chemotypes rather than an effect exclusively associated with myristicin.

Such an interpretation is consistent with the general behavior of EOs rich in lipophilic phenylpropanoids and monoterpenes, whose antimicrobial activity has frequently been associated with their ability to partition into lipid bilayers, perturb membrane organization and increase permeability [50]. However, because the whole EOs contain multiple volatile constituents, the faster response observed for **As** cannot be attributed exclusively to apiol and may also reflect additive or synergistic interactions with other abundant constituents, particularly *β*-phellandrene and *γ*-terpinene.

Taken together, these observations suggest that apiol and myristicin may converge on membrane perturbation as a common antimicrobial effect, while differences in their chemical structures and in the overall composition of the corresponding EOs may influence the kinetics and magnitude of membrane damage.

### 2.5. Antibiofilm Activity

**Figure 8:**
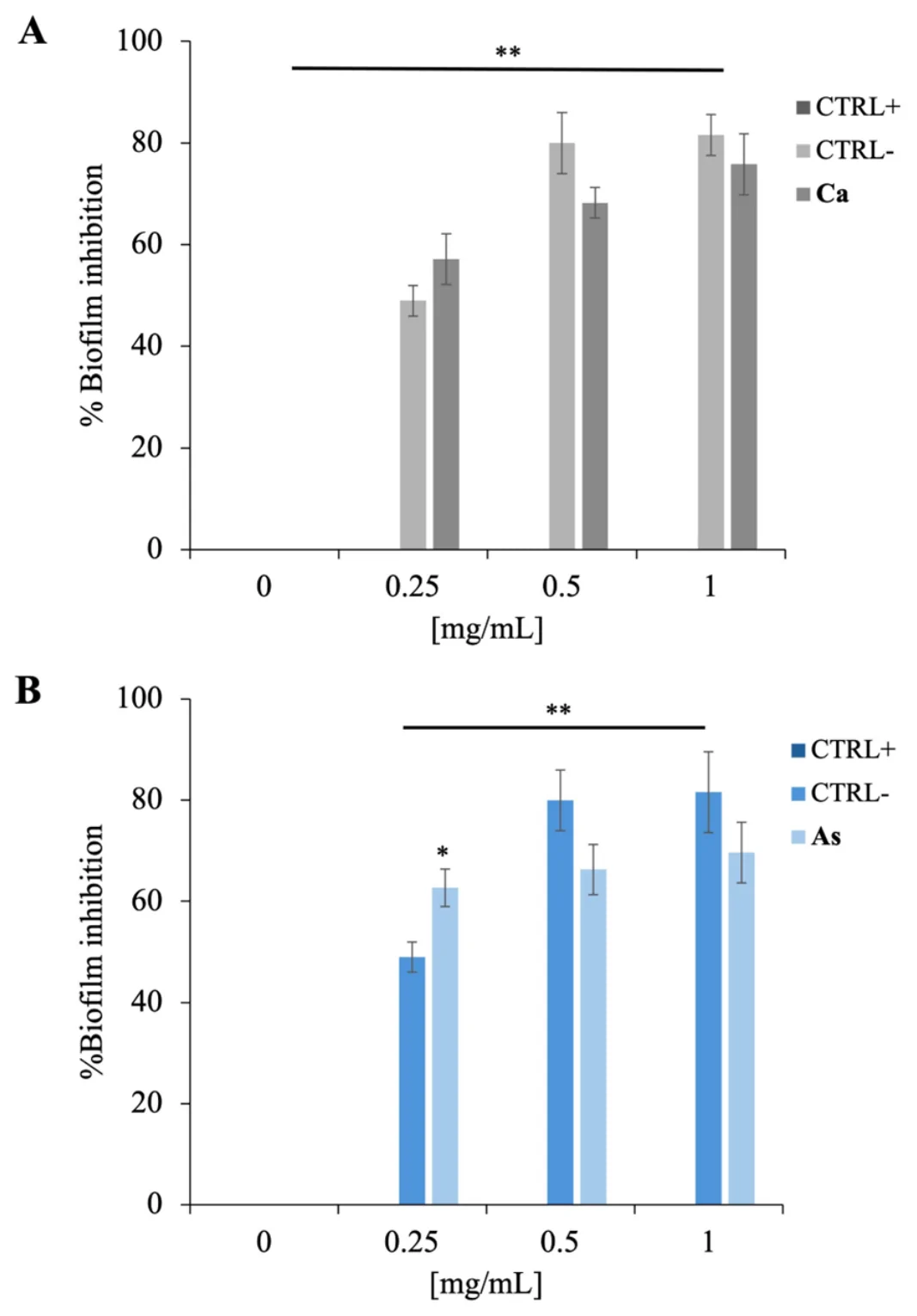
Inhibition of biofilm formation by *Athamanta* EOs chemotypes on *Mycobacterium smegmatis*. Panel (**A**): Effect of **Ca** (previously published data); Panel (**B**): **As** inibion activity. CTRL+ represents untreated biofilm-forming cells, while CTRL− represents cells treated with kanamycin. Data are expressed as the mean ± standard deviation (SD) of three independent experiments. Statistical analysis was performed using a two-tailed Student’s *t*-test for paired data compared to the untreated control (* *p* < 0.05; ** *p* < 0.01). **As**: *Athamanta sicula* apiol-rich EO from Roccamena; **Ca**: *A. sicula* myristicin-rich from Caltabellotta.

Since biofilm formation represents one of the primary factors involved in the persistence of bacterial infections and the development of tolerance mechanisms toward antimicrobial agents, the ability of **As** and **Ca** to interfere with this process was also evaluated and compared. As shown in Figure 8, both oils demonstrated a marked dose-dependent activity.

In Panel A, relating to **Ca**, biofilm inhibition progressively increases as the EO concentration increases, rising from approximately 57% at 0.25 mg/mL up to values exceeding 70% at concentrations of 0.5 and 1 mg/mL. A similar trend was observed for **As** (Panel B). In this case as well, **As** showed a significant reduction in biofilm formation even at the lowest concentration tested (0.25 mg/mL), with an inhibition of approximately 63%, which is higher than that observed for **Ca** at the same concentration. At concentrations of 0.5 and 1 mg/mL, the activity was substantially comparable to that of **Ca**, with inhibition values close to 66–70%. Therefore, while **Ca** (Figure 8A) shows a progressive increase in activity with increasing concentration, **As** (Figure 8B) appears to maintain a slightly higher antibiofilm capacity even at lower doses. This suggests a potentially greater efficacy during the initial phases of cell adhesion and extracellular matrix formation. Overall, the results indicate that the antibiofilm activity of the two chemotypes is largely conserved despite the profound differences in the chemical compositions. However, **As** showed a tendency to exert greater inhibition at the lowest concentrations, suggesting that apiol may interestingly contribute to interfering with the mechanisms involved in the early stages of biofilm development. These data confirm that the capacity to counteract biofilm formation represents a common functional characteristic of *Athamanta* EOs and support the hypothesis that the predominant phenylpropanoids, despite belonging to different chemotypes, share biological mechanisms capable of limiting bacterial adhesion and the organization of sessile microbial communities. This property is particularly interesting from an applicative perspective, considering the central role of biofilms in chronic infectious processes and in the reduced sensitivity to conventional antibiotics.

### 2.6. Antioxidant Activity

**Figure 9:**
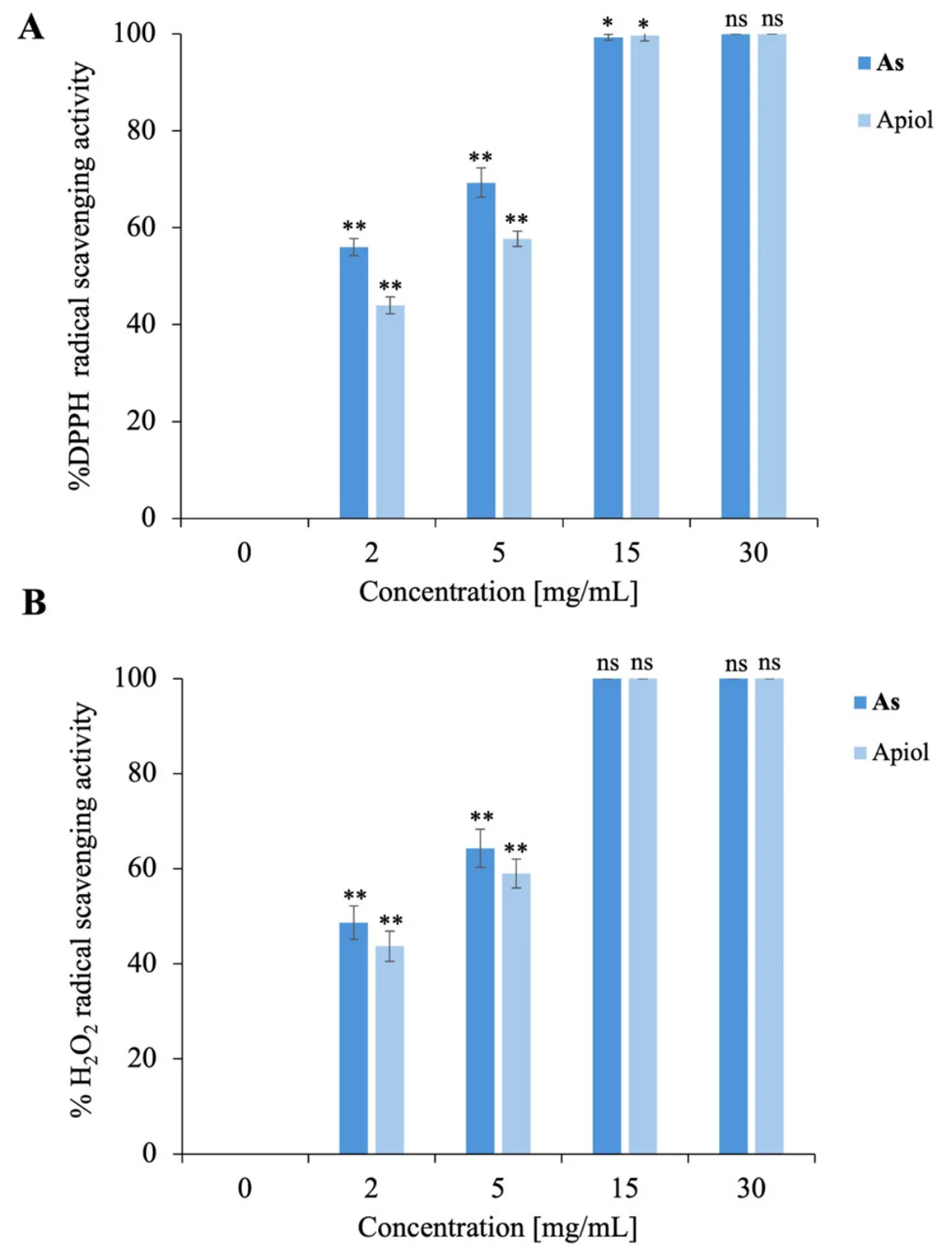
Antioxidant activity of **As** and apiol. Panel (**A**): DPPH assay; Panel (**B**): H_2_O_2_ scavenging assay. **As** and apiol were tested at concentrations ranging from 0 to 30 mg/mL. Data represent the average of three independent experiments. Statistical analysis was performed relative to the standard by two-tailed paired *t*-test. *p*-vaule (ns not significant; * *p* ≤ 0.05; ** *p* < 0.01). **As**: *Athamanta sicula* apiol-rich EO from Roccamena; **Ca**: *A. sicula* myristicin-rich from Caltabellotta.

The antioxidant activity of **As** and **Ca** was evaluated using two complementary assays: DPPH and H_2_O_2_ scavenging. The results obtained for **As** were compared with those previously reported for the myristicin-rich chemotype (**Ca**), in order to assess whether the different phenylpropanoid composition influences the trend of the antioxidant response (Figure 9).

In the DPPH assay, the two chemotypes (**As** and **Ca**) showed a clearly different trend at low concentrations. **Ca** had previously exhibited a lower initial activity, both for the while EO and for myristicin, with limited scavenging values at 2 and 5 mg/mL [50]. Conversely, **As** (Figure 9A) showed a more marked response, both for whole sample and for apiol, starting from the first concentrations tested. In particular, the **As** reached approximately 56% scavenging activity at 2 mg/mL and about 69% at 5 mg/mL, indicating greater DPPH scavenging efficiency at low concentrations. Apiol showed approximately 48% scavenging activity at 2 mg/mL and about 59% at 5 mg/mL, compared to 40% for myristicin at the same concentration [50]. However, as the concentration increased, both chemotypes and their individual components achieved very high antioxidant activity values, reaching near-complete scavenging activity at 30 mg/mL. A similar trend was observed in the H_2_O_2_ assay. In this case too, **As** (Figure 9B) showed higher activity at low concentrations compared to the profile previously observed for **Ca** [50]. **As** reached approximately 49% scavenging at 2 mg/mL and about 64% at 5 mg/mL, and apiol deviated very little from these values, while both showed complete neutralization of hydrogen peroxide at concentrations of 15 and 30 mg/mL. **Ca**, by contrast, had shown a more gradual response at low concentrations, reaching maximum activity only at the highest concentrations [50]. The comparison between the two chemotypes thus suggests that the different composition does not alter the maximum antioxidant capacity, which is high in both samples, but mainly influences the intensity of the response at low concentrations. In particular, **As** appears to show greater initial scavenging efficiency compared to **Ca**, as do the individual components themselves. The results obtained were further confirmed by calculating the IC_50_ values (Table 7).

**As** showed lower IC_50_ values than **Ca,** in both the DPPH and H_2_O_2_ assays, indicating greater scavenging efficiency even at low concentrations. A similar trend was observed when comparing the two major phenylpropanoids: apiol exhibited IC_50_ values of 2.0 ± 0.7 and 2.3 ± 0.7 mg/mL in the DPPH and H_2_O_2_ assays, respectively, which were lower than those observed for myristicin (6.2 ± 1.2 and 7.1 ± 0.5 mg/mL, respectively).

The lower IC_50_ values observed for both the whole EO and its major constituent are consistent with the results of the scavenging assays, suggesting that **As** has a greater ability to neutralize radical species at low concentrations than the myristicin-rich chemotype (**Ca**).

The parallel behavior observed for the whole EOs and their predominant phenylpropanoids is particularly noteworthy. The greater scavenging efficiency of the apiol-rich **As** was accompanied by lower IC_50_ values for apiol compared with myristicin, supporting an important contribution of the predominant phenylpropanoid to the antioxidant profile of **As**. Nevertheless, the antioxidant activity of the whole EO cannot be attributed exclusively to apiol, since **As** is a complex phytochemical mixture in which other constituents and interactions among major and minor components may also contribute to the overall scavenging response.

This difference may reflect the different efficiencies of the two phenylpropanoids in electron-transfer or hydrogen-atom-donation processes.

Antioxidant properties have also been reported for EOs of other Apiaceae species. For example, Di Girolamo et al. [67] demonstrated that the EO of *Pastinaca sativa* subsp. *sativa*, characterized by a markedly different volatile profile dominated by octyl acetate and octyl butanoate, reduced H_2_O_2_-induced intracellular ROS accumulation in Caco-2 cells. Although these results cannot be directly compared quantitatively with the DPPH and H_2_O_2_ cell-free assays used in the present study, they provide additional evidence that chemically distinct Apiaceae EOs may exhibit antioxidant properties through different experimental models [67].

Overall, these results indicate that, among the biological activities investigated in the present study, antioxidant activity is likely the property most strongly influenced by chemotypic differentiation among *Athamanta* chemotypes. Although both chemotypes maintain a high scavenging capacity at higher concentrations, the apiol-rich chemotype (**As**) exhibited a more efficient antioxidant response at lower concentrations than **Ca**. These findings highlight how variations in the phytochemical profile may translate into measurable functional differences.

## 3. Materials and Methods

### 3.1. Sampling Sites

Five populations of *A. sicula* were investigated in this study: four from Sicily (Roccamena, Caltabellotta, Monte Pellegrino, and the Madonie Mountains), and one from north-ern Algeria (Didouche Mourad). The geographic coordinates of the Didouche Mourad site in Algeria [48] and of the Monte Pellegrino and Madonie sites in Sicily (Italy) [49,50,51] were obtained by georeferencing locality information reported in the literature from textual descriptions of place names. Although this approach provides lower spatial accuracy than GPS-based records, it remains the most widely adopted method for georeferencing species occurrence record. For the Caltabellotta and Roccamena sites, geographic coordinates were collected directly in the field (Table 8). Chemical data for the Didouche Mourad, Madonie and Monte Pellegrino populations were taken from the literature [48,49,51], whereas those for Caltabellotta and Roccamena were obtained by our research group ([50] and present study). The available information on collection date and plant material is summarized in Table 8. Since the literature studies analyzed different organs, the values used in Table 3 correspond to the mean of the flower, stem and leaf EOs for Monte Pellegrino [51] and to the leaf EO for Madonie [49], whereas the fruit EO of the latter population was not considered, to ensure comparability with the vegetative and flowering material analyzed for the other populations.

From a bioclimatic perspective, according to the classification proposed by Rivas-Martínez et al. [68], all investigated sites fall within the Mediterranean pluviseasonal oceanic bioclimate, as also reported for the Mediterranean Region by Gianguzzi and Bazan [69]. The corresponding thermotypic and ombrotypic horizons were determined from monthly climatic data obtained from the CHELSA dataset [53], using the bioclimatic indices and threshold values proposed by Rivas-Martínez et al. [68]. The indices were calculated using a custom Python tool implemented in QGIS. The resulting classification of each sampled population is reported in Table 9.

Climatic data were obtained from CHELSA version 2.1 (Climatologies at High resolution for the Earth’s Land Surface Areas) [53,55]. CHELSA provides gridded climate data at a horizontal resolution of 30 arc-seconds (~1 km), derived from a mechanistic, terrain-informed downscaling of atmospheric reanalysis data (ERA5 in version 2.1). Temperature is downscaled using lapse rates derived from vertical atmospheric temperature profiles, whereas precipitation is downscaled by accounting for orographic effects, including windward–leeward exposure and valley exposition, and is subsequently bias-corrected against station observations of the Global Precipitation Climatology Centre (GPCC) [55,70]. Owing to this approach, CHELSA performs better than purely statistical interpolation products in topographically complex regions, particularly for precipitation [55], which makes it suitable for the heterogeneous relief of Sicily and northern Algeria. In this study, the bioclimatic variables for the reference period 1981–2010 (CHELSA_bioXX_1981–2010_V.2.1) were used at their native resolution, without resampling. These variables represent long-term climatological means and thus describe the climatic regime of each site, rather than the weather conditions of the sampling year. Weather conditions of the growing seasons preceding sampling (daily mean air temperature and total precipitation from 1 September to the sampling date) were obtained for the grid cells including each site from the ERA5 reanalysis at 0.25° resolution [57], retrieved through the Open-Meteo Historical Weather API [58], and expressed as anomalies relative to the 1981–2010 means for the same period of the year. For the literature populations, for which only the month of collection is reported [49,51], the 15th day of that month was used as the reference date.

The investigated populations span three Mediterranean thermotypic belts. Monte Pellegrino falls within the upper thermomediterranean horizon; Caltabellotta and Roccamena within the lower mesomediterranean horizon; Didouche Mourad within the upper mesomediterranean horizon; and the Madonie population within the lower supramediterranean horizon. Most sites belong to the lower subhumid ombrotype, whereas the Madonie population is classified as lower humid. Despite their geographic and bioclimatic differentiation, all investigated populations of *A. sicula* occur on carbonate substrates, predominantly consisting of limestone and dolomitic limestone [71,72].

### 3.2. Isolation of EO

Fresh aerial parts (flowers, leaves, and stems, total 900 g) of Roccamena *A. sicula* (**As**) were subjected to hydro-distillation for 3 h, according to the standard procedure described in the European Pharmacopoeia [73]. The sample, dehydrated with sodium sulfate (Na_2_SO_4_), was stored at 4 °C in a freezer in a vial equilibrated under an N_2_ atmosphere. Samples yielded 0.12% of EO. Plant material of the Roccamena population was collected on 2 May 2024, at full flowering stage. A voucher specimen, identified by G. Bazan, was deposited in the *Herbarium Mediterraneum Panormitanum* (PAL) of the University of Palermo (voucher No. 109828).

### 3.3. GC-MS Analysis

Analysis of EO was carried out according to the procedure reported by Vaglica et al. [74]. GC-MS analysis was performed using a Shimadzu QP 2010 plus equipped with an AOC-20i autoinjector (Shimadzu, Kyoto, Japan) gas chromatograph equipped with a capillary column (DB-5 MS) of 30 m × 0.25 mm i.d., film thickness 0.25 μm, and a data processor. The oven program was as follows: temperature was held at 40 °C for 5 min, then increased at a rate of 2 °C/min up to 260 °C, then isothermal for 20 min. Helium was used as carrier gas (1 mL min^−1^). The injector and detector temperatures were set at 250 and 290 °C, respectively. One μL of essential oil solution (3% essential oil/hexane *v*/*v*) was injected in split mode 1:50; MS range 40–600. The settings were as follows: ionization voltage, 70 eV; electron multiplier energy, 2000 V; transfer line temperature, 295 °C; solvent delay, 3 min.

Linear retention indices (LRIs) were calculated using a mixture of pure *n*-alkanes (C_8_–C_30_), and all the peaks’ compounds were identified by comparison with MS and by comparison of their relative retention indices with WILEY275, NIST 17, AD-AMS, and FFNSC2 libraries.

### 3.4. Isolation of Apiol, Spectroscopical Analysis (NMR) and Chemicals

^1^H- spectrum was recorded as reported in Castagliuolo et al. [75]. Deuterated chloroform (CDCl_3_) and *n*-alkanes (C_8_–C_30_) were purchased from Sigma-Aldrich (Milan, Italy). **As** (~100 mg) was fractionated, using a flash silica gel column (Biotage^®^ Sfär Silica, Uppsala, Sweden). Columns were typically run using a gradient method, beginning with 100% petroleum ether and gradually increasing the polarity using ethyl acetate. A 99:1 (*v*/*v*) petroleum ether-ethyl acetate mixture was used for isolation of 37 mg of apiol. The NMR spectrum was recorded on a Bruker Avance II instrument (400 MHz for ^1^H-NMR analysis, Bruker, Billerica, MA, USA). Deuterated chloroform (CDCl_3_) was used as solvent with the peaks at 7.27 ppm as references for ^1^H-NMR spectrum.

### 3.5. Statistical Analysis

Given the limited number of sampled populations, the statistical analysis was designed as an exploratory approach aimed at describing the direction and magnitude of the relationships between environmental variables and the chemical composition of the essential oil, rather than at providing formal inferential evidence. The analytical dataset consisted of five sampled sites: Caltabellotta, Monte Pellegrino, Didouche Mourad, Madonie, and Roccamena. For each site, bioclimatic variables extracted from CHELSA raster layers and the MaxEnt climatic suitability value were associated with the relative abundance (%) of the main volatile compounds detected in the chemical profile.

The environmental matrix included annual mean temperature (BIO1), temperature seasonality (BIO4), maximum temperature of the warmest month (BIO5), minimum temperature of the coldest month (BIO6), annual precipitation (BIO12), precipitation seasonality (BIO15), precipitation of the driest quarter (BIO17), precipitation of the warmest quarter (BIO18), and MaxEnt suitability. The chemical matrix included the relative abundance of apiol, myristicin, germacrene B, *β*-phellandrene and *γ*-terpinene.

Pairwise correlations were calculated between each environmental variable and each volatile compound. Three complementary correlation metrics were used: Pearson’s correlation coefficient, Spearman’s rank correlation coefficient and Kendall’s tau. Pearson’s coefficient was used to describe linear relationships between climatic variables and compound abundance. Spearman’s coefficient was used to evaluate monotonic relationships based on the rank order of the sites, thereby reducing the influence of the absolute magnitude of the values. Kendall’s tau was included as an additional non-parametric measure of concordance between the ordering of sites according to environmental and chemical variables.

For each pairwise comparison, the observed correlation coefficient was calculated using the five site values. Because of the very small sample size, conventional parametric significance tests were not considered appropriate. Therefore, exact permutation-based *p*-values were calculated by considering all possible permutations of the five observed values. Since the number of sampled sites was five, a total of 5! = 120 possible permutations were evaluated for each correlation. The two-sided permutation *p*-value was estimated as the proportion of permuted correlation coefficients whose absolute value was greater than or equal to the absolute value of the observed coefficient.

The analysis was therefore interpreted in terms of effect size, direction of association and consistency among the different correlation metrics. Correlations were not used to infer statistically significant causal relationships, but rather to identify preliminary ecological patterns linking climatic differentiation, MaxEnt suitability and chemotypic variability among populations. In particular, strong and consistent correlations across Pearson, Spearman and Kendall coefficients were considered as potential indicators of biologically meaningful trends, whereas isolated correlations driven by a single population or by compounds detected in only one site were interpreted with caution.

No correction for multiple testing was applied, as the analysis was exploratory and hypothesis-generating. The resulting patterns were used to support an ecological interpretation of the observed chemical differentiation among populations and to identify candidate climatic variables potentially associated with variation in the relative abundance of the main volatile compounds.

Considering that *Athamanta sicula* is a chasmophytic species mainly occurring on limestone cliffs and rocky outcrops, the distribution analysis at the geographical scale was aimed at defining the climatic suitability of the species rather than providing a fine-scale description of its microhabitat. For this purpose, MaxEnt was used to estimate climatically suitable areas for the species and to evaluate the role of the main bioclimatic factors in shaping its potential distribution.

Eight bioclimatic variables derived from the CHELSA dataset were considered: BIO1—annual mean temperature, BIO4—temperature seasonality, BIO5—maximum temperature of the warmest month, BIO6—minimum temperature of the coldest month, BIO12—annual precipitation, BIO15—precipitation seasonality, BIO17—precipitation of the driest quarter, and BIO18—precipitation of the warmest quarter. These variables were selected because they represent annual climatic conditions, thermal extremes, water availability and seasonal climatic stress, which are key determinants of the geographical distribution of plant species, particularly in Mediterranean environments. All variables were taken from the CHELSA V2.1 bioclimatic dataset for the period 1981–2010 (CHELSA_bioXX_1981–2010_V.2.1) and used at their native resolution of 30 arc-seconds [53].

Occurrence data for *Athamanta sicula* at the global scale were obtained from georeferenced records available in online databases, with particular reference to GBIF (https://doi.org/10.15468/dl.tvhymt, accessed on 16 June 2026), and were integrated with the sampling sites used for metabolomic analyses. The initial dataset, consisting of 119 records, was subjected to a revision and cleaning procedure by removing records with inaccurate, inconsistent or duplicated geographic information. The final dataset used for modeling included 79 occurrence localities, including the five sites investigated for metabolite analyses.

The model calibration area was delimited from the species occurrence localities by constructing a Minimum Convex Polygon. To include an area that was ecologically and biogeographically accessible to the species, the polygon was subsequently expanded by applying a 210 km buffer. This area was used as the geographic background for the generation of random points and for the extraction of available environmental conditions. The preparation, checking and alignment of the environmental raster layers and occurrence data were carried out in QGIS to ensure spatial consistency between the climatic layers and the presence records used for subsequent modeling.

The model was calibrated using MaxEnt v.3.4.1, applying the default software settings and enabling response curves and the jackknife test. Calibration was performed using the occurrence records of *Athamanta sicula* and the eight continuous bioclimatic variables. Model validation was conducted using 10-fold cross-validation, with 10 replicate runs. Predictive performance was evaluated using the area under the receiver operating characteristic curve (AUC) and the omission rate. The mean test AUC across replicate runs was 0.907, with a standard deviation of 0.035, indicating good discriminatory ability of the model. The relative contribution of environmental variables was assessed using percent contribution, permutation importance, jackknife analysis and response curves.

The climatic suitability map, showing the spatial distribution of areas predicted as suitable for *Athamanta sicula*, was produced in ArcGIS Pro 3.7 from the MaxEnt output raster.

### 3.6. Bacterial Strains

The antimicrobial activity of **As** and apiol were evaluated against different strains Gram-negative such as: *Escherichia coli* DH5α, *Pseudomonas aeruginosa* PAO1 ATCC15692, and *Salmonella typhimurium* ATCC14028; Gram-positive such as: *Staphylococcus aureus* ATCC6538P, *Bacillus subtilis* AZ54, *Listeria monocytogenes* ATCC 19115, and *Mycobacterium smegmatis* MC^2^155 and the yeast *Candida albicans* ATCC14053. *E. coli* and *S. aureus* were used as model strains.

### 3.7. Determination of Minimum Inhibitory Concentrations (MIC)

The antimicrobial activity of **As** and apiol was evaluated by determining the minimum inhibitory concentrations (MICs) against selected bacterial strains using the broth microdilution method, according to CLSI guidelines [76].

Bacterial cultures (5 × 10^5^ CFU/mL), grown overnight, were added to 96-well microplates containing 195 μL of Mueller–Hinton broth (CAM-HB; Difco, Becton, Dickinson and Company. Franklin Lakes, NJ, USA). Samples diluted in 50% DMSO were tested at concentrations ranging from 1 to 30 mg/mL, based on the relative proportion of each component in the EO. Plates were incubated at 37 °C for 24 h.

The MIC was assessed by measuring the optical density at 600 nm using a microplate reader, defined as the lowest sample concentration that completely inhibited visible growth at OD 600 nm. Untreated cells served as negative controls, whereas ampicillin and 50% DMSO were used as positive controls.

To confirm MIC results, 15 μL of resazurin solution (0.01%) was added to each well, followed by incubation at 37 °C for 30 min. The redox-dependent color change was recorded by measuring absorbance at 600 nm [50].

### 3.8. N-Phenyl Naphthylamine (NPN) Assay

The ability of **As** to permeabilize the bacterial outer membrane (OM) was assessed by means of the 1-N-phenylnaphthylamine (NPN) uptake assay, adapted from the method described by Jia et al. [77] with minor modifications.

*E. coli* cultures were incubated overnight under standard growth conditions, then collected by centrifugation, rinsed with PBS, and resuspended in 5 mM HEPES buffer (pH 7.2) until reaching an optical density of 0.5 ± 0.02 at 600 nm [78]. For each experimental condition, 50 µL of the test sample (used at concentrations corresponding to the MIC and twice the MIC) was mixed with 100 µL of the bacterial suspension in black 96-well fluorescence microplates. Subsequently, 50 µL of a 40 µM NPN solution was added. Control wells consisted of bacterial cells and NPN in HEPES buffer without **As**. Fluorescence was measured at excitation and emission wavelengths of 350 nm and 420 nm, respectively, using a microplate fluorimeter. The increase in fluorescence intensity was used as an indicator of OM permeabilization.

### 3.9. Fluorescence-Based Viability Assay Using DAPI and PI

The integrity of the bacterial inner membrane (IM) was evaluated by fluorescence microscopy using DAPI (4′,6-diamidino-2-phenylindole dihydrochloride; Sigma-Aldrich, Milan, Italy) and propidium iodide (PI; Sigma-Aldrich, Milan, Italy), following the method described by Di Girolamo et al. [67].

Briefly, 100 μL of *E. coli* and *S. aureus* suspensions were incubated at 37 °C for 4 h under shaking, in the presence or absence of **As** and apiol at their respective MIC values (for *E. coli*: 25 and 10 mg/mL; for *S. aureus*: 30 and 15 mg/mL). Following incubation, 10 μL of each sample was combined with a staining solution containing DAPI (1 μg/mL) and PI (20 μg/mL) and incubated in the dark. Fluorescence images were obtained using an Olympus BX51 microscope (Olympus, Tokyo, Japan) equipped with a filter set specific for DAPI detection (excitation/emission: 358/461 nm). The exposure time for DAPI/PI double staining was fixed at 1000 ms. Image acquisition was performed using an Olympus DP70 digital camera.

### 3.10. Inhibition of Biofilm Development Assays

The antibiofilm activity of **As** was evaluated against *Mycobacterium smegmatis* biofilms using the crystal violet (CV) colorimetric assay, as described by Castagliuolo et al. [79].

Biofilms were grown in 24-well plates and incubated at 37 °C for 3 days with or without **As** at concentrations ranging from 0 to 1 mg/mL. Untreated cells were used as positive controls, while kanamycin-treated cultures served as negative controls.

Following incubation, the biofilm biomass was stained with crystal violet, and absorbance was measured at 570 nm using a Multiskan microplate reader (Thermo Electron Corporation, Waltham, MA, USA). To correct for differences in planktonic growth, results were normalized by calculating the ratio between OD_570_ (biofilm biomass) and OD_600_ (planktonic cell density). The percentage of biofilm inhibition was then determined by comparing the normalized OD values of treated samples to those of untreated controls according to the formula:

Biofilm formation (%) = [(OD_570_/OD_600_)treated/(OD_570_/OD_600_)control] × 100

where (OD_570_/OD_600_) treated represents the normalized biofilm value in the presence of **As** or antibiotics; (OD_570_/OD_600_) control represents the normalized biofilm value of the untreated control; and after:

Biofilm inhibition (%) = (100-% Biofilm formation)

### 3.11. DPPH and H_2_O_2_ Scavenging Capacity Assay

The antioxidant capacity of **As** and apiol, was evaluated through two complementary assays: DPPH radical scavenging and hydrogen peroxide scavenging, following the methodology described by Napolitano et al. For both assays, a range of concentrations (0–30 mg/mL) was prepared in a final reaction volume of 1 mL [80].

In the DPPH assay, a freshly prepared 0.1 mM DPPH solution in 100% methanol was adjusted to an initial absorbance of ≤1.0. Samples were incubated with the DPPH solution at room temperature for 30 min, after which the decrease in absorbance at 517 nm was measured.

For the hydrogen peroxide scavenging assay, fresh hydrogen peroxide solution (50 mM potassium phosphate buffer, pH 7.0; 0.036% *w*/*w* H_2_O_2_) was used. Samples at varying concentrations were incubated with 1 mL of this solution at room temperature for 30 min. After centrifugation to remove any particulate matter, the absorbance of the supernatant was measured at 240 nm to assess the residual hydrogen peroxide. The scavenging percentage was determined by:

H_2_O_2_ and DPPH scavenged (%) = (1 − Abs/AC) × 100

where AC represents the absorbance of the solution without the sample, and Abs is the absorbance following incubation with the sample. To account for turbidity and potential physical quenching due to the immiscibility of the EO in methanol, each sample was first read before DPPH addition (T_0_), and again after 30 min of incubation (T_30_). The corrected absorbance value (AS) was calculated as T_30_ − T_0_, ensuring the removal of background absorbance caused by the oil phase.

As positive control ascorbic acid was included treated under the same experimental conditions.

Together, these assays provided a comprehensive evaluation of the antioxidant potential against both stable free radicals and reactive oxygen species.

## 4. Conclusions

The present study indicates that *Athamanta sicula* comprises distinct essential oil chemotypes, whose geographical distribution is associated with the macroclimatic conditions of the sites, leading to the occurrence of distinct essential oil chemotypes within the same species. In particular, the Apiol-rich chemotype from Roccamena markedly differs from the previously described Myristicin-rich chemotype from Caltabellotta, suggesting that climatic differentiation among sites may act as a selective factor on chemotypic composition, although the relative contributions of genetic differentiation and phenotypic plasticity remain to be assessed.

Despite these pronounced compositional differences, both chemotypes maintained comparable antimicrobial, antibiofilm and antioxidant activities, indicating that chemotypic variation does not compromise the overall biological functionality of the essential oil. Nevertheless, the apiol-rich chemotype displayed enhanced activity against *Pseudomonas aeruginosa* and a more rapid antioxidant response at lower concentrations, suggesting that chemotypic differentiation may also generate subtle functional specialization.

Overall, these findings support the hypothesis that chemotypic differentiation may represent an adaptive response, allowing *A. sicula* essential oils to maintain their defensive functions under different environmental conditions, a hypothesis that remains to be tested. From an applied perspective, understanding the relationship between climatic conditions, phytochemical composition and biological activity provides a valuable framework for the identification and selection of chemotypes with specific bioactive properties. From an applied perspective, understanding the relationship between climatic conditions, phytochemical composition and biological activity provides a valuable framework for the identification and selection of chemotypes with specific bioactive properties. This knowledge may contribute to evaluating the potential of Athamanta sicula essential oils as natural antimicrobial and antioxidant agents for pharmaceutical, biotechnological and food preservation applications. However, considering the high relative abundance of apiol (47.5%) in the Roccamena chemotype, its potential toxicological implications should also be carefully considered. Although the present biological assays were performed taking into account the relative proportion of apiol in the essential oil, the promising in vitro activities reported here do not establish its safety for food or pharmaceutical use. Therefore, specific toxicological studies and the definition of safe exposure levels will be necessary before practical applications of the apiol-rich chemotype can be considered.

## Figures and Tables

**Table 1:** Chemical composition (%) of *Athamanta sicula* L. EO (**As**) collected in Roccamena, Sicily, Italy.

No.	Compound ^a^	LRI ^b^	LRI ^c^	Area (%) ^d^
1	*β*-Thujene	925	920	0.1
2	α-Pinene	930	931	0.3
3	Camphene	946	947	0.1
4	Sabinene	969	969	0.2
5	*β*-Pinene	988	982	1.1
6	*α*-Phellandrene	999	998	2.2
7	*β*-Phellandrene	1034	1032	32.7
8	*cis-β*-Ocimene	1037	1034	1.5
9	*trans-β*-Ocimene	1046	1044	0.8
10	γ-Terpinene	1057	1060	6.3
11	4-Terpineol	1167	1173	0.1
12	*α*-Terpineol	1183	1188	0.1
13	Bornyl acetate	1265	1261	0.2
14	α-Copaene	1349	1361	0.2
15	*β*-Caryophyllene	1389	1394	0.6
16	*α*-Bergamotene	1406	1409	0.0
17	Humulene	1424	1430	0.1
18	*γ*-Muurolene	1447	1449	0.0
19	*γ*-Elemene	1466	1455	0.8
20	Zingiberene	1471	1473	0.6
21	*δ*-Cadinene	1490	1495	1.0
22	Elemicin	1529	1540	0.8
23	Apiol	1667	1679	47.5
	Monoterpene Hydrocarbons			45.3
	Oxygenated Monoterpenes			0.4
	Sesquiterpene Hydrocarbons			3.3
	Phenylpropanoids			48.3
	Total			97.3

^a^ Components listed in order of elution on a DB-5 MS apolar column; ^b^ LRIs based on literature (https://webbook.nist.gov/ accessed on 20 July 2026), ^c^ Experimental LRIs on a DB-5 MS apolar column; ^d^ Content is the relative area percentage of single compound obtained from TIC mass chromatogram of the EO sample.

**Table 2:** Climatic characterization and MaxEnt suitability values of the sampled populations of *A. sicula*.

Sites	BIO01	BIO04	BIO05	BIO06	BIO12	BIO15	BIO17	BIO18	Suitability
Caltabellotta	15.05	5.24	26.25	5.05	851	62.6	32.2	89.5	0.999877
Madonie	11.15	5.54	22.85	0.75	928	51.7	72.5	72.5	0.990690
MontePellegrino	16.55	5.13	26.95	7.75	765	58.0	36.6	100.8	0.997453
Roccamena	16.95	6.04	31.45	4.75	781	55.5	36.7	36.7	0.351656
DidoucheMourad	14.35	6.67	31.65	0.35	782	55.1	46.3	46.3	0.345193

BIO01 = annual mean temperature (°C); BIO04 = temperature seasonality; BIO05 = maximum temperature of the warmest month (°C); BIO06 = minimum temperature of the coldest month (°C); BIO12 = annual precipitation (mm); BIO15 = precipitation seasonality; BIO17 = precipitation of the driest quarter (mm); BIO18 = precipitation of the warmest quarter (mm). Suitability values that represent the output of the MaxEnt model. All bioclimatic variables are CHELSA V2.1 climatological means for the reference period 1981–2010, extracted at the native resolution of 30 arc-seconds (~1 km) [53,54]; following the CHELSA V2.1 specification, BIO17 and BIO18 are expressed as mean monthly precipitation within the respective quarter (mm month^−1^).

**Table 3:** Relative abundance (%) of the main volatile compounds detected in the sampled populations of *Athamanta sicula*. The table reports the percentage values for each site investigated.

Sites	Apiol	Myristicin	Germacrene B	*β*-Phellandrene	*γ*-Terpinene
Caltabellotta (**Ca**)	2.2	62.2	0	12.2	6.9
Madonie	0	97.6	0	0	0
Monte Pellegrino	84.5	4.5	0	0	0
Roccamena (**As**)	47.5	0	0	32.7	6.3
Didouche Mourad	4.9	0	88.5	0	0

**As**: *Athamanta sicula* apiol-rich EO from Roccamena; **Ca**: *A. sicula* myristicin-rich from Caltabellotta.

**Table 4:** Exploratory pairwise correlations between bioclimatic variables, MaxEnt suitability and the relative abundance of the main volatile compounds detected in the sampled populations of *Athamanta sicula*.

Climate Variable	Metabolite	Pearson r	Pearson Exact *p*	Spearman rho	Spearman Exact *p*	Kendall tau-a
BIO01	Apiol	0.718	0.150	0.800	0.133	0.600
BIO01	Myristicin	−0.810	0.083	−0.564	0.400	−0.500
BIO01	Germacrene B	−0.112	0.800	−0.354	0.800	−0.200
BIO01	*β*-Phellandrene	0.554	0.350	0.671	0.300	0.500
BIO01	*γ*-Terpinene	0.453	0.400	0.447	0.500	0.300
BIO04	Apiol	−0.334	0.592	−0.200	0.783	−0.200
BIO04	Myristicin	−0.446	0.500	−0.564	0.400	−0.300
BIO04	Germacrene B	0.832	0.200	0.707	0.400	0.400
BIO04	*β*-Phellandrene	0.122	0.850	0.112	1.000	0.100
BIO04	*γ*-Terpinene	−0.147	0.850	−0.112	1.000	−0.100
BIO05	Apiol	0.203	0.725	0.600	0.350	0.400
BIO05	Myristicin	−0.880	0.050	−0.975	0.033	−0.900
BIO05	Germacrene B	0.572	0.400	0.707	0.400	0.400
BIO05	*β*-Phellandrene	0.465	0.550	0.112	1.000	0.100
BIO05	*γ*-Terpinene	0.220	0.800	−0.112	1.000	−0.100
BIO06	Apiol	0.802	0.183	0.500	0.450	0.400
BIO06	Myristicin	−0.340	0.583	0.205	0.733	0.100
BIO06	Germacrene B	−0.603	0.400	−0.707	0.400	−0.400
BIO06	*β*-Phellandrene	0.277	0.750	0.224	0.800	0.100
BIO06	*γ*-Terpinene	0.342	0.400	0.335	0.600	0.300
BIO12	Apiol	−0.670	0.175	−1.000	0.017	−1.000
BIO12	Myristicin	0.982	0.050	0.667	0.267	0.500
BIO12	Germacrene B	−0.323	0.800	0.000	1.000	0.000
BIO12	*β*-Phellandrene	−0.247	0.650	−0.112	1.000	−0.100
BIO12	*γ*-Terpinene	−0.051	0.950	0.112	1.000	0.100
BIO15	Apiol	0.124	0.842	0.400	0.517	0.400
BIO15	Myristicin	−0.132	0.817	−0.051	1.000	0.100
BIO15	Germacrene B	−0.205	0.600	−0.354	0.800	−0.200
BIO15	*β*-Phellandrene	0.165	0.700	0.447	0.500	0.300
BIO15	*γ*-Terpinene	0.593	0.350	0.671	0.300	0.500
BIO17	Apiol	−0.456	0.475	−0.400	0.517	−0.400
BIO17	Myristicin	0.642	0.200	0.051	1.000	−0.100
BIO17	Germacrene B	0.049	1.000	0.354	0.800	0.200
BIO17	*β*-Phellandrene	−0.453	0.400	−0.447	0.500	−0.300
BIO17	*γ*-Terpinene	−0.588	0.250	−0.671	0.300	−0.500
BIO18	Apiol	0.260	0.733	0.100	0.950	0.000
BIO18	Myristicin	0.353	0.550	0.564	0.400	0.300
BIO18	Germacrene B	−0.466	0.600	−0.354	0.800	−0.200
BIO18	*β*-Phellandrene	−0.520	0.350	−0.447	0.500	−0.300
BIO18	*γ*-Terpinene	−0.162	0.750	−0.112	1.000	−0.100
Suitability	Apiol	0.045	0.933	0.000	1.000	0.000
Suitability	Myristicin	0.663	0.200	0.649	0.333	0.400
Suitability	Germacrene B	−0.612	0.400	−0.559	0.800	−0.300
Suitability	*β*-Phellandrene	−0.469	0.700	−0.177	1.000	−0.100
Suitability	*γ*-Terpinene	−0.124	1.000	0.177	1.000	0.100

BIO01 = annual mean temperature (°C); BIO04 = temperature seasonality; BIO05 = maximum temperature of the warmest month (°C); BIO06 = minimum temperature of the coldest month (°C); BIO12 = annual precipitation (mm); BIO15 = precipitation seasonality; BIO17 = precipitation of the driest quarter (mm month^−1^); BIO18 = precipitation of the warmest quarter (mm month^−1^); Suitability = climatic suitability value estimated by the MaxEnt model. Bioclimatic variables are CHELSA V2.1 climatological means for the period 1981–2010 [53,55].

**Table 5:** Minimum inhibitory concentrations (MICs) of the EOs obtained from **Ca** and **As** against different bacterial strains, determined by the broth microdilution method. Values are expressed in mg/mL and represent the mean of three independent experiments. Data for **Ca** were previously published and are included here for comparative purposes.

	MIC [mg/mL]	
Strains	Ca ^1^	As
*E. coli*	25	25
*S. aureus*	30	30
*P. aeruginosa*	25	17
*L. monocytogenes*	29	30
*S. Typhimurium*	24	28
*B. subtilis*	26	23
*M. smegmatis*	30	30

^1^ Previously published data [50]. **As**: *Athamanta sicula* apiol-rich EO from Roccamena; **Ca**: *A. sicula* myristicin-rich from Caltabellotta.

**Table 6:** Minimum inhibitory concentrations (MICs) of the major phenylpropanoids myristicin and apiol against different bacterial strains, determined by the broth microdilution method. Values are expressed in mg/mL and represent the mean of three independent experiments. Data for myristicin were previously published and are included here for comparative purposes.

	MIC [mg/mL]	
Strains	Myristicin ^1^	Apiol
*E. coli*	15	10
*S. aureus*	16	15
*P. aeruginosa*	14	9
*L. monocytogenes*	17	15
*S. Typhimurium*	15	14
*B. subtilis*	16	16
*M. smegmatis*	20	18

^1^ Previously published data [50].

**Table 7:** IC_50_ values of the antioxidant activity **Ca** and **As**, their respective major constituents (myristicin and apiol), and ascorbic acid (positive control), determined by DPPH and H_2_O_2_ radical scavenging assays. Values are expressed as mg/mL (mean ± standard deviation) of three independent experiments. Data for **Ca** were previously published and are included here for comparative purposes.

	IC_50_ [mg/mL] ± SD				
	Ca ^1^			As	
Sample	DPPH	H_2_O_2_	Sample	DPPH	H_2_O_2_
EO	8.5 ± 1.9	7.8 ± 0.3	**As**	1.7 ± 0.3	2 ± 0.4
Myristicin	6.2 ± 1.2	7.1 ± 0.5	Apiol	2 ± 0.7	2.3 ± 0.7
Ascorbic acid	0.03 ± 0.03	0.04 ± 0.05	Ascorbic acid	0.03 ± 0.03	0.04 ± 0.05

^1^ Previously published data [50]. **As**: *Athamanta sicula* apiol-rich EO from Roccamena; **Ca**: *A. sicula* myristicin-rich from Caltabellotta.

**Table 8:** Geographic coordinates and elevation of the five *Athamanta sicula* sampling sites investigated in Sicily and northern Algeria.

Site	Longitude	Latitude	Elevation (m., a.s.l.)	Collection Date	Plant Material Analyzed	Ref.
Didouche Mourad	6.655476	36.40331	972	June 2009	Leaves ^a^	[48]
Roccamena	13.1308	37.85544	333	2 May 2024	Aerial parts ^b^	Present work
Caltabellotta	13.16852	37.57745	554	10 May 2025	Aerial parts ^b^	[50]
Monte Pellegrino	13.34355	38.16792	205	May 2006	Flowers, stems, leaves ^c^	[51]
Madonie	14.00768	37.88323	1275	July 2000	Leaves ^d^	[49]

^a^ Reported as fresh aerial parts in the Abstract and as fresh leaves in the Methods of [48]; ^b^ flowers, leaves and stems collected at flowering; ^c^ EOs obtained separately from each organ, the mean of the three organs being used in Table 3; ^d^ the fruit EO analyzed in [49] was not considered. Coordinates and elevation of the Didouche Mourad, Monte Pellegrino and Madonie sites were obtained by georeferencing the locality descriptions reported in the literature.

**Table 9:** Thermotypic and ombrotypic classification of the investigated *Athamanta sicula* populations according to the bioclimatic classification of Rivas-Martínez et al. [68].

Sites	Thermotype	Ombrotype
Caltabellotta	Lower Mesomediterranean	Lower Subhumid
Monte Pellegrino	Upper Thermomediterranean	Lower Subhumid
Didouche Mourad	Upper Mesomediterranean	Lower Subhumid
Madonie	Lower Supramediterranean	Lower Humid
Roccamena	Lower Mesomediterranean	Lower Subhumid

## Data Availability

The original contributions presented in this study are included in the article. Further inquiries can be directed to the corresponding authors.
